# Low efficiency of large volcanic eruptions in transporting very fine ash into the atmosphere

**DOI:** 10.1038/s41598-019-38595-7

**Published:** 2019-02-05

**Authors:** Mathieu Gouhier, Julia Eychenne, Nourddine Azzaoui, Arnaud Guillin, Mathieu Deslandes, Matthieu Poret, Antonio Costa, Philippe Husson

**Affiliations:** 1 Université Clermont Auvergne, CNRS, IRD, OPGC, Laboratoire Magmas et Volcans, F-63000 Clermont-Ferrand, France; 2 Laboratoire de Mathématiques Blaise Pascal, UMR 6620 CNRS & UCA, Aubière, France; 3 VAAC Toulouse, Météo France, Toulouse, France; 4 Istituto Nazionale di Geofisica e Vulcanologia, Sezione di Bologna, Bologna, Italy

## Abstract

Volcanic ash clouds are common, often unpredictable, phenomena generated during explosive eruptions. Mainly composed of very fine ash particles, they can be transported in the atmosphere at great distances from the source, having detrimental socio-economic impacts. However, proximal settling processes controlling the proportion (ε) of the very fine ash fraction distally transported in the atmosphere are still poorly understood. Yet, for the past two decades, some operational meteorological agencies have used a default value of ε = 5% as input for forecast models of atmospheric ash cloud concentration. Here we show from combined satellite and field data of sustained eruptions that ε actually varies by two orders of magnitude with respect to the mass eruption rate. Unexpectedly, we demonstrate that the most intense eruptions are in fact the least efficient (with ε = 0.1%) in transporting very fine ash through the atmosphere. This implies that the amount of very fine ash distally transported in the atmosphere is up to 50 times lower than previously anticipated. We explain this finding by the efficiency of collective particle settling in ash-rich clouds which enhance early and en masse fallout of very fine ash. This suggests that proximal sedimentation during powerful eruptions is more controlled by the concentration of ash than by the grain size. This has major consequences for decision-makers in charge of air traffic safety regulation, as well as for the understanding of proximal settling processes. Finally, we propose a new statistical model for predicting the source mass eruption rate with an unprecedentedly low level of uncertainty.

## Introduction

Volcanic ash clouds generated by explosive eruptions are distally transported in the atmosphere up to distances ranging from a few hundreds to thousands of kilometres from the vent. They are mainly composed of the finest ash fraction which survives proximal sedimentation, and referred to as very fine ash (<32 µm) following the physical volcanology-derived terminology of explosive eruptions^1^. However, in some cases coarser particles can reach distal location as recently demonstrated during moderate Icelandic eruptions^2^. Very fine ash can have damaging effects on aircraft, hence having detrimental impact on air traffic safety as demonstrated by air traffic disruption during Eyjafjallajökull^3^ and Cordón del Caulle^4^ eruptions. Distal airborne very fine ash represent only a fraction of the total amount of solid particles (referred to as tephra) injected into the volcanic plume column above the crater. Here we examine this partitioning (*ε;* given in percentage) as the ratio between the very fine ash flux transported in distal clouds (*Q*_*a*_) estimated from satellite-based infrared measurements, and the total flux of tephra emitted at the source (*Q*_*s*_) inferred from ground studies of tephra deposits. The latter is also referred to as the Mass Eruption Rate (MER). The ratio *ε* quantifies the volcanic ash removal efficiency in proximal areas, which is critical for constraining ash sedimentation processes during the early stages of cloud dispersal, as well as for predicting the ash clouds properties as they are advected around the globe.

We compiled a database of 22 eruptions of various magnitudes and intensities carefully selected from remarkably well documented case studies in the published records (Table 1). They are characterized by distinct eruption styles describing the dynamics and phenomenology of the explosive activity. Eruption styles can be defined from various classifications using different parameters^5,6^. In our case, we adopted the most recent one^6^ for the following reasons: (i) it uses the MER (equivalent to *Q*_*s*_) and the volcanic ash plume height (*H*) as input parameters; (ii) it allows individual eruption phases to be easily requalified; and (iii) permits a real-time first order classification of eruptive events, which is useful for operational applications. Therefore, we distinguish sustained eruptions (9 Small/Moderate, 7 Subplinian and 4 Plinian styles) defined by quasi-steady discharge conditions (i.e., with a duration of tephra emission much longer than the time necessary to reach the neutral buoyancy level) from transient eruptions corresponding to unsteady impulsive explosions (i.e., 2 Vulcanian style).

**Table 1:** Eruptive parameters for the 22 eruptions of our dataset.

Volcano	Explosive Phase selected	Duration (s)	Fallout deposit mass (Kg)	±Error (Kg)	Cloud mass (Kg)	±Error (Kg)	Plume Height (km)	Q_s_ (kg/s)	Q_a_ (kg/s)	ε (%)	±Error	Style
Pinatubo	Climactic phase on 15–16/06/1991	3.24E + 04^(1)^	5.7E + 12^(2)^	1.4E + 12	5.0E + 10^(3)^	2.5E + 10	40^(1)^	1.8E + 08	1.5E + 06	0.9	0.6	Plinian^(1,2)^
Kelut	Full eruption 13/02/2014	1.08E + 04^(4)^	6.5E + 11^(5)^	1.6E + 11	7.4E + 08^(4)^	3.7E + 08	20^(4)^	6.0E + 07	6.9E + 04	0.1	0.1	Plinian^(5)^
El Chichon	Phases B and C on 04/04/1982	3.96E + 04^(6)^	8.7E + 11^(7)^	2.2E + 11	6.5E + 09^(8)^	3.3E + 09	30^(8)^	2.2E + 07	1.6E + 05	0.7	0.5	Plinian^(6)^
Hudson	Full eruption 12–15/08/1991	2.27E + 05^(9)^	3.9E + 12^(10)^	9.8E + 11	2.9E + 09^(11)^	1.5E + 09	18^(9,11)^	1.7E + 07	1.3E + 04	0.1	0.0	Plinian^(10)^
Sarychev Peak	Subplinian events on 14–15/06/2009	5.94E + 04^(12)^	4.0E + 11^(12)^	1.0E + 11	5.4E + 08^(12)^	2.7E + 08	20^(12)^	6.7E + 06	9.1E + 03	0.1	0.1	Subplinian*
Cordon Caulle	Climactic phase on 4–5/06/2011	8.64E + 04^(13)^	4.5E + 11^(13)^	1.1E + 11	7.0E + 08^(14)^	3.5E + 08	12.2^(14)^	5.2E + 06	8.1E + 03	0.2	0.1	Subplinian^(13)^
Grimsvotn	Subplinian phase on 22/05/2011	1.91E + 05^(15)^	7.0E + 11^(15,16)^	1.8E + 11	4.9E + 08^(17)^	2.5E + 08	20^(17,18)^	3.7E + 06	2.6E + 03	0.1	0.0	Subplinian^(16)^
Mt. Spurr	Full eruption 16/09/1992	1.30E + 04^(19)^	3.9E + 10^(20)^	9.8E + 09	6.1E + 08^(21)^	3.1E + 08	13.9^(21)^	3.0E + 06	4.7E + 04	1.6	1.0	Subplinian^(20)^
Mt. Spurr	Full eruption 18/08/1992	1.25E + 04^(19)^	3.6E + 10^(20)^	9.0E + 09	4.2E + 08^(21)^	2.1E + 08	13.7^(21)^	2.9E + 06	3.4E + 04	1.2	0.8	Subplinian^(20)^
Redoubt	Explosive events 1 to 5 on 22–23/03/2009	5.22E + 03^(22)^	1.4E + 10^(22)^	3.5E + 09	1.7E + 09^(23)^	8.5E + 08	15^(22)^	2.7E + 06	3.3E + 05	12.1	8.0	Vulcanian^(22)^
Mt. Spurr	Full eruption 27/06/1992	1.46E + 04^(19)^	3.1E + 10^(20)^	7.8E + 09	4.4E + 08^(21)^	2.2E + 08	14.5^(21)^	2.1E + 06	3.0E + 04	1.4	0.9	Subplinian^(20)^
Lascar	Full eruption 04/1993	1.73E + 05^(24)^	3.5E + 11^(21,25)^	8.6E + 10	4.8E + 09^(21)^	2.4E + 09	21^(24)^	2.0E + 06	2.8E + 04	1.4	0.9	Subplinian*
Anatahan	Explosive phases on 10–11/05/2003	3.24E + 04^(26)^	3.8E + 10^(27)^	9.6E + 09	1.3E + 09^(26)^	6.5E + 08	12^(26)^	1.2E + 06	4.0E + 04	3.4	2.2	Small/Moderate^(27)^
Chaiten	Full eruption: 2–8/05/2008	6.05E + 05^(28)^	1.7E + 11^(28)^	4.3E + 10	8.0E + 08	4.0E + 08	19^(28)^	2.8E + 05	1.3E + 03	0.5	0.3	Subplinian^(28)^
Hekla	Phase I + 8 hrs phase II on 26/02/2000	4.21E + 04^(29)^	1.0E + 10^(30)^	2.5E + 09	1.0E + 08^(29)^	5.0E + 07	11^(29)^	2.4E + 05	2.4E + 03	1.0	0.7	Small/Moderate*
Soufrière Hills	Full eruption: 26/09/1997	3.60E + 03^(31)^	5.5E + 08^(32)^	1.4E + 08	5.4E + 07^(33)^	2.7E + 07	11.3^(33)^	1.5E + 05	1.5E + 04	9.8	6.4	Vulcanian^(32)^
Ruapehu	Full eruption: 17/06/1996	3.60E + 04^(34)^	4.2E + 09^(35)^	1.1E + 09	2.9E + 08^(36)^	1.5E + 08	8.5^(36)^	1.2E + 05	8.1E + 03	6.9	4.6	Small/Moderate*
Eyjafjallajökull	Phase I/III on 14–19/04 & 05–18/05/2010	1.90E + 06^(37)^	2.0E + 11^(38)^	4.9E + 10	8.3E + 09^(39)^	4.2E + 09	9^(38)^	1.0E + 05	4.4E + 03	4.2	2.8	Small/Moderate
Etna	Full eruption: 28/10/2002	2.16E + 04^(40)^	1.1E + 09^(40)^	2.6E + 08	1.1E + 07^(41)^	5.7E + 06	6^(42)^	4.9E + 04	5.3E + 02	1.1	0.7	Small/Moderate^(40)^
Popocatepetl	Climactic events on 10/03/1996	2.16E + 04^(43)^	5.3E + 08^(44)^	1.3E + 08	1.5E + 07^(43)^	7.5E + 06	9^(44)^	2.4E + 04	6.9E + 02	2.8	1.9	Small/Moderate*
Etna	Full eruption: 27/10/2002	3.60E + 04^(40)^	8.7E + 08^(40)^	2.2E + 08	2.4E + 07^(42)^	1.2E + 07	6^(42)^	2.4E + 04	6.6E + 02	2.7	1.8	Small/Moderate^(40)^
Etna	Full eruption: 24/11/2006	2.16E + 04^(45)^	1.0E + 08^(46)^	2.5E + 07	3.6E + 06^(47)^	1.8E + 06	1.5^(45)^	4.6E + 03	1.7E + 02	3.6	2.4	Small/Moderate^(46)^

The eruptions were selected in the dataset providing that quality published data existed in the literature on the mass of the fallout deposit (derived from field analyses) and the mass of the very fine ash cloud (derived from satellite-based measurements). The plume height above the vent comes from different types of observational data (remote-sensing, visual estimations, etc.). *Q*_*s*_ and *Q*_*a*_ are calculated as the fallout deposit mass and very fine ash cloud mass, respectively, divided by the duration of the eruption phase. ε is calculated as the ratio of *Q*_*a*_ over *Q*_*s*_. Eruption styles have been determined using *Q*_*s*_ and the plume height^6^ and are in agreement with related published records. *Eruption styles for explosive phases that have not been published and which may differ from existing classifications made for full eruption or a different phase.

The database comprises satellite-based infrared measurements of *Q*_*a*_ inferred from the extinction properties of ash using the split-window method^7,8^, except for the May, 2011 Grimsvötn and October 27, 2002 Etna eruptions, whose measurements were carried out using hyperspectral sounders. This technique provides vertical column densities as a mass per unit area, and allows total mass of very fine ash to be retrieved from integration of ash-bearing pixels over the whole cloud surface. Then, the average value of *Q*_*a*_ can be calculated by simply dividing the total mass by the duration of ash emission. Most of the data come from Low-Earth Orbiting (LEO) platforms, hence allowing image acquisition ∼10 hours on average after the start of the eruption. The large Instantaneous Field Of View (IFOV) of these sensors usually allows the observation of the whole cloud from a single or a few images. The extent of the cloud determines the ash emission duration and the timing of the image acquisition allows us to identify the explosive phase within the eruption chronology. The inversion of thermal infrared measurements is particularly relevant for the characterization of the very fine ash content of volcanic clouds because it allows the retrieval of particles in the size range 1–32 µm (at 2σ for a lognormal distribution^2^). However, some known uncertainties remain from (i) the split-window technique leading to false detection and missed ash-bearing pixels^9^ as well as from (ii) the limits of the validity domain within the Mie scattering theory^2,8^. Overall uncertainty associated with satellite-based measurements was estimated to be in the range ±40–60%^2^.

The average value of *Q*_*s*_ was calculated from the published mass of tephra deposited on the ground, divided by the duration of the explosive phase. Importantly, *Q*_*a*_ and *Q*_*s*_ refer to the same period of explosive activity and can thus be reliably compared. The temporal concordance required between these 2 parameters explains the relatively low number of eruptions finally selected. The deposit masses selected in this study (Table 1) were calculated by integrating the mass decay rate of the fallout deposit or integrating the thinning rate of the fallout deposit^10,11^. These methods are sensitive to the quality and density of field data, to the mathematical function chosen (e.g., exponential, power-law, Weibull) to represent their spatial variations and to the distal extrapolation limit. Indeed, individual measurements of tephra thickness or mass are extrapolated at greater distances than the maximum sampling, allowing the finest ash fraction to be accounted for, and the total mass of tephra estimated. Thus, *Q*_*s*_ represents the average MER of the total grain size distribution at the source vent and is given with an uncertainty of ±10–40%^12,13^. Note that uncertainties on each individual eruption for both satellite and ground deposits retrievals have not been systematically published or inferred, but we specifically selected eruptions for which the measurements errors should be low (e.g. no clouds in the atmosphere, no erosion of the deposit, sampling performed hours to days following the eruptions, etc.). The related error on *ε* has been calculated from the average bulk uncertainties of *Q*_*a*_ and *Q*_*s*_ and reported for each eruption in Table 1.

## Results

### Source-to-atmosphere very fine ash partitioning

**Figure 1:**
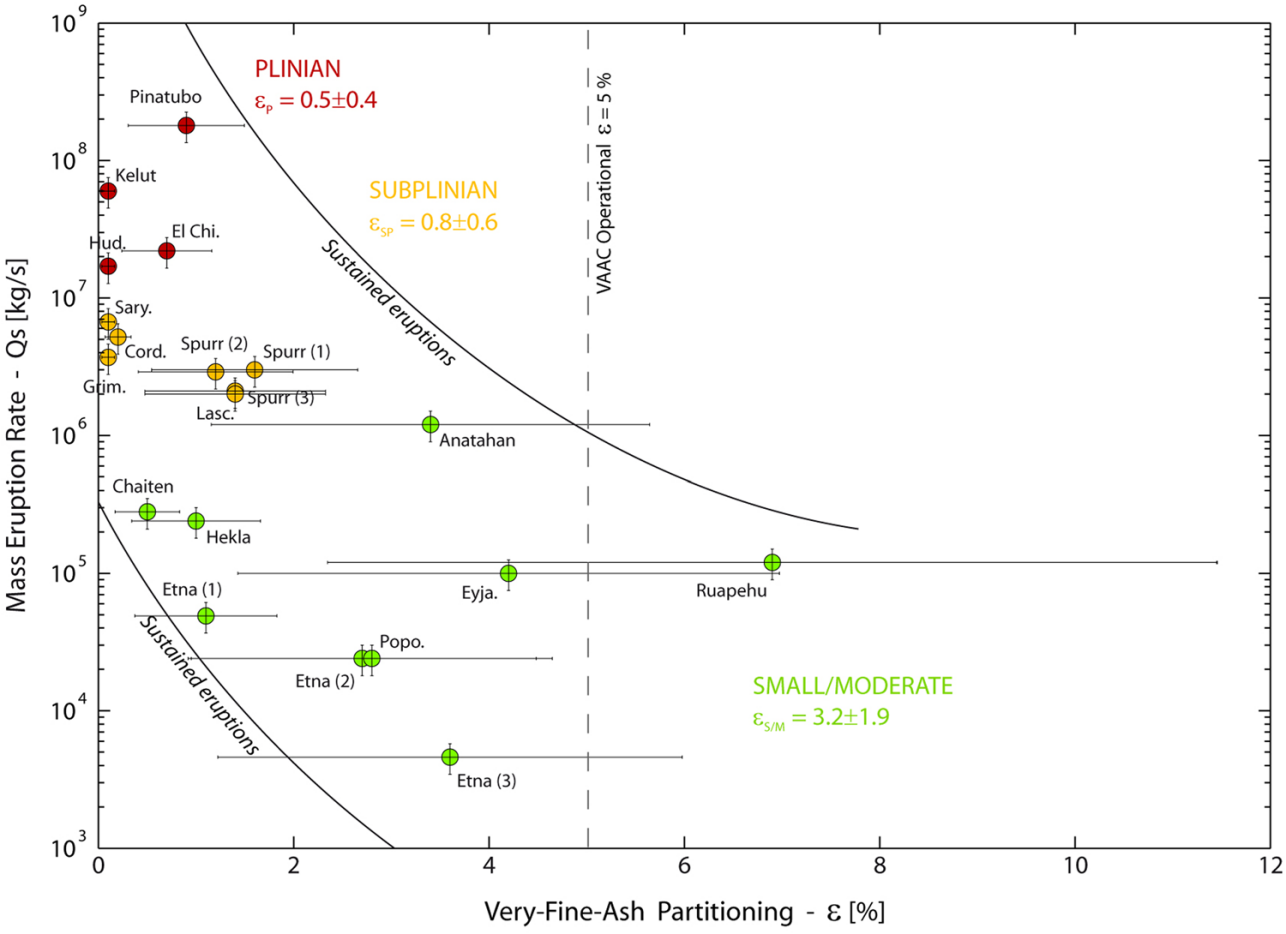
Style-derived volcanic ash partitioning of sustained eruptions. Mass erupting rate (*Q*_*s*_ in kg/s) as a function of the partitioning coefficient *ε* (*Q*_*a*_/*Q*_*s*_ in %) for the 20 sustained eruptions of our dataset (see Table 1 for details about the eruptions). *ε* is the ratio between the very fine ash flux transported in distal clouds (*Q*_*a*_) and the flux of tephra in the plume (*Q*_*s*_) also referred to as MER. It quantifies the volcanic very fine ash removal efficiency. The sustained eruptions cluster following their eruption style (Plinian, Subplinian, Small/Moderate). This plot shows that *ε* of sustained eruptions scales with *Q*_*s*_, and spans about two orders of magnitude. The main trend shows that *ε* increases with decreasing MER. This indicates that very fine ash removal from ash-rich plumes (Plinian and Subplinian style) is more efficient than from plumes containing coarser tephra (Small/Moderate style). Error bars are plotted from average bulk uncertainties given for fallout deposit and cloud masses (see Table 1 for detailed error values). The vertical dashed line represents the current VAAC operational partitioning coefficient used by to forecast the atmospheric path of very fine ash clouds. The eruption-dependant partitioning coefficients for each eruption style (*ε*_*P*_, *ε*_*SP*_, *ε*_*S/M*_) have also been reported.

We show that *ε* of sustained eruptions spans a wide range of values, from 0.1% (e.g., Plinian Kelut 2014 eruption) to 6.9% (Small/Moderate Ruapehu 1996 eruption; Table 1). Fine ash removal from Plinian eruptions is thus about two orders of magnitude more efficient than that from Small/Moderate ones. Remarkably, the variation of the partitioning coefficient is not arbitrary. From Fig. 1, *ε* decreases with increasing MER, with respect to eruption styles. The four Plinian eruptions selected have large MER (1.7 × 10^7^ < *Q*_*s*_ < 1.8 × 10^8^ kg/s). They all produced copious amount of volcanic ash, as for the 1980 Mount St Helens, and the 1982 El Chichón eruptions, for which the mass fraction of ash smaller than 63 µm represents ∼50% of the total mass of tephra emitted^14,15^. Such high fine ash contents are related to efficient magma fragmentation processes, occurrence of phreato-magmatic episodes, and contribution of ash elutriated from pyroclastic density currents (PDC) forming co-PDC plumes^16^. However, they all exhibit a very small proportion of distal very fine ash, as shown by the weak partitioning coefficient range (0.1 < *ε* < 0.9%), and fall in a well delimited area in Fig. 1. To explain this observation, we suggest that early enhanced fallout in proximal regions makes the actual proportion of very fine ash transported in distal clouds much lower than expected. This highlights the critical role played by collective settling mechanisms, occurring preferentially in ash-rich plumes, which enhance the sedimentation rate of tephra regardless of grain size. Such mechanisms include aggregation^17,18^, gravitational instabilities^19^, diffusive convection^20^, particle-particle interactions^21^, and wake-capture effects^22^. These are inferred to be key processes controlling the early depletion of ash-rich plumes, which cannot be explained by individual particle settling. Aggregation efficiency, in particular, has been identified^23,24^ to be proportional to a power greater than two of ash concentration. This means that the higher the fine ash concentration the more important the aggregation efficiency, which is in agreement with the observations made in our study.

Collective settling mechanisms, allow en masse sedimentation of particles of different sizes, which explains the significant amount of fine ash as well as the poor grain sorting sometimes observed in proximal tephra fallout deposits of large Plinian events. The fallout deposit from the 18 May 1980 eruption of Mount St. Helens (MSH80) for instance, shows both a poor grain sorting in proximal locations^25,26^ and an increase of mass and thickness at distances >300 km^27^ demonstrating rapid removal of fine ash from the plume. Different enhanced sedimentation processes have been invoked and successfully tested to explain these observations, including aggregation^28^, and hydrometeor formation^14^. Subplinian eruptions, although less powerful than Plinian ones, remain very explosive and capable of efficient fragmentation, also leading to the formation of ash-rich plumes. For example, the August and September 1992 Mt. Spurr eruptions produced fallout deposits with fine ash contents reaching 30% and 40% of the total mass of tephra, respectively^29^. The origin of this fine ash is discussed, and could be related to heterogeneities in the source magma or secondary particle fragmentation in the volcanic conduit or eruption column^30^. They have MER in the range 0.28–6.7 × 10^6^ kg/s and still exhibit low partitioning coefficient, although spanning a wider range of values (0.1 < *ε* < 1.6%), hence implying collective settling mechanisms to be at work. The Mt Spurr fallout deposits also show an increase of mass and thickness at distances >150 km from the source, which can be explained by collective settling mechanisms including aggregation, topographic effects and gravitational instabilities^30^. Small/Moderate eruptions are drastically different from Plinian and Subplinian. The eruption explosivity and the MER are much weaker. The plume column height is generally lower and the fine ash fraction of the size distribution at the source vent is much smaller. Consequently, Small/Moderate eruptions do not produce ash-rich plumes, and enhanced sedimentation in proximal regions is limited, resulting in larger partitioning coefficients (0.5 < *ε* < 6.9%). The wide range of *ε* values for Small/Moderate eruptions reflects the heterogeneity of grain size and concentration of the associated plumes. But, this can also be explained by the natural complexity of some long-lasting eruptions. This is the case, in particular, for the Eyjafjallajökull 2010 eruption displaying multiple and discontinuous phases of explosive activity with varying intensity.

The inverse relationship between *ε* and the MER shows that for very powerful eruptions, the proximal sedimentation is mainly controlled by the concentration of fine ash. This suggests that above a given threshold of the fine ash volume fraction, collective mechanisms dominate over individual particle settling, and conversely. Assessment of this threshold is very difficult as proximal measurements (i.e., in the first tens of kilometres from the source vent) of airborne volcanic ash concentration are scarce. Indeed, satellite-based retrievals are usually impossible due to the opacity of the cloud. But, radar instruments operating at larger wavelengths are able to provide volcanic ash concentration within proximal cloud. The comparison of ash concentration at various distances, hence using various techniques, should bring precious information on early depletion processes and about sedimentation rate evolution. As an example, proximal measurements carried out in the first two hours after the MSH80 Plinian eruption by a 23-cm wavelength radar^31^ give an ash cloud concentration of ∼8.5 g/m^3^. In this region of the cloud we expect sedimentation rate to be high with a significant contribution of collective particle settling mechanisms. As a comparison, distal measurements carried out by satellite-based infrared sensors on the Kelut 2014 Plinian eruption^32^ give an ash cloud concentration 3 orders of magnitude lower (maximum value ∼9 mg/m^3^). In this region of the cloud, the sedimentation rate is very low and the individual particle settling is most likely to prevail.

### Ash cloud hazards and operational response

The partitioning parameter *ε* is crucial in operational volcanic risk mitigation, as it is required as input for ash-cloud-dispersal models used by several VAACs responsible for global air traffic safety. Given that satellite images are not systematically available, the VAACs need rapid parameterization schemes to predict *Q*_*a*_, and to provide frequent and reliable up-to-date forecast maps of atmospheric ash concentration during volcanic crises^33^. With this aim, VAACs (such as London and Toulouse) have typically used a poorly constrained default *ε* value of 5%^34^ to forecast the concentration of very fine ash composing distal ash clouds following *Q*_*a*_ = *ε* × *Q*_*s*_. However, as demonstrated in Fig. 1, the fraction of very fine ash that survives proximal settling varies by ∼2 orders of magnitudes (0.1% > *ε* > 6.9%) with respect to the MER. Therefore, a constant partitioning value cannot be used, even as first order estimate for operational purposes. Note that *Q*_*s*_ is needed, and usually obtained operationally from top plume height estimates following a power-law relationship^35^ between the two parameters. The reliability of *Q*_*s*_ estimate from this method is discussed later in this work and compared with our satellite-based prediction model of *Q*_*s*_ (see next section). Here we propose a new operational eruption-style-dependant parameterization of *ε* using the mean values for Plinian (*ε*_*P*_ = 0.5%), Subplinian (*ε*_*SP*_ = 0.8%), and Small/Moderate (*ε*_*S/M*_ = 3.2%) eruptions (Fig. 1). This parameterization is easily implementable in ash-cloud-dispersal models, allowing operational use by the VAACs. Also, the choice of the correct partitioning parameter to be used during the course of an eruption is not difficult. The phenomenology as well as the real-time assessment of *Q*_*s*_ will be particularly useful to discriminate the eruption style. In some cases, the eruptive history at each volcanic target can also be helpful. Our assessed values of *ε* significantly depart from the default 5% value used by the VAACs (Fig. 1), and the resulting differences will propagate into the modelled ash cloud concentrations.

**Figure 2:**
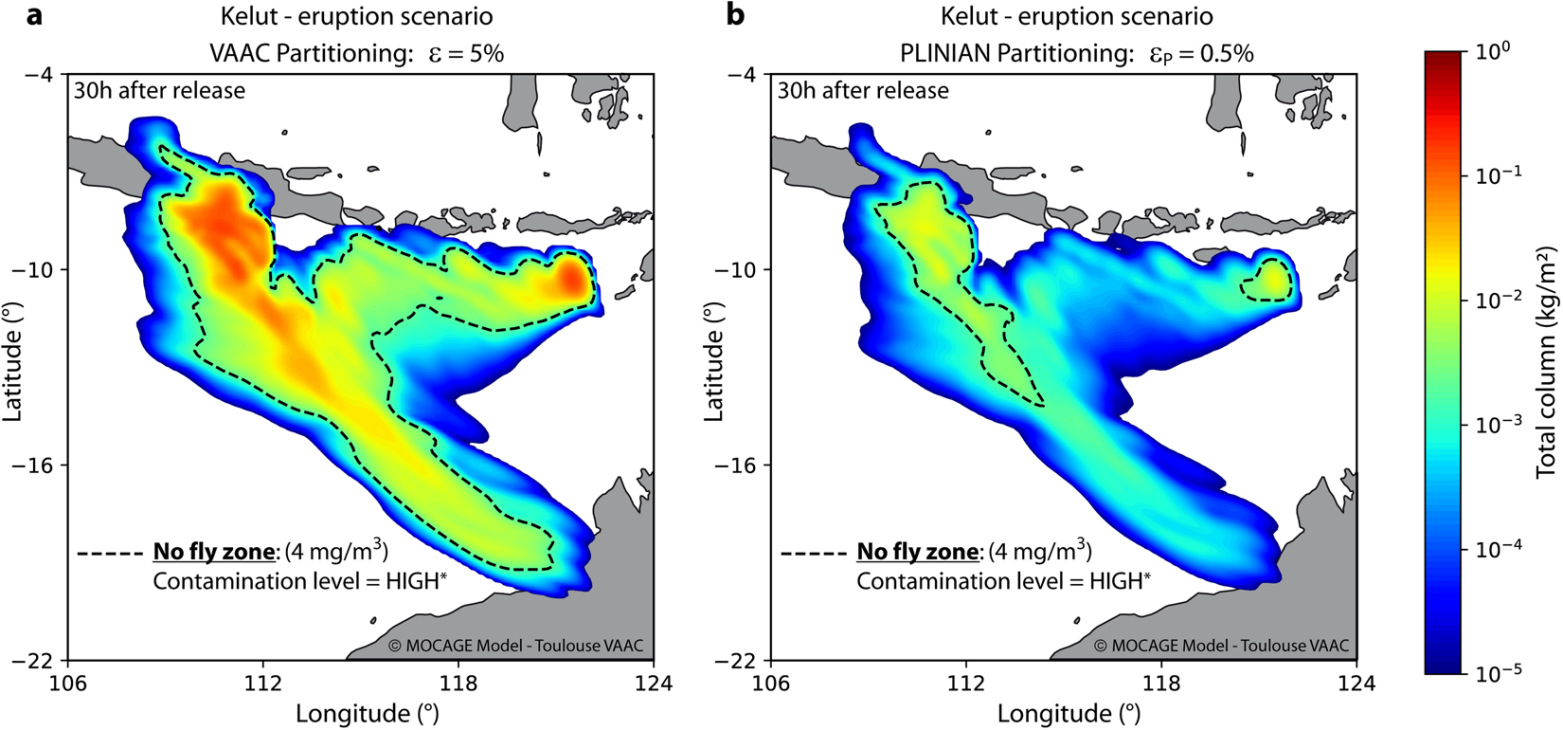
Ash cloud concentration simulations during Plinian eruptions. The two simulations are produced by the volcanic ash-cloud-dispersal model MOCAGE of the Toulouse VAAC based on the Plinian eruption of Kelut the 13 February, 2014 (Supplementary Information Table 1), using different partitioning coefficients and present-day meteorological data. (**a**) Simulation of ash dispersion in the atmosphere at Kelut volcano 30 hours after the eruption, using the VAAC-default operational *ε* value of 5%. (**b**) Same simulation conditions and scenario, but using the Plinian *ε* value established in this study at 0.5%. The extent of the No-Fly zone (4 mg/m^3^ for an ash cloud 500-m thick*) is much larger for the VAAC-default *ε*, yielding a maximum concentration one order of magnitude higher. *The threshold at 4 mg/m^3^ was first established by the European Commission after the Eyjafjallajökull 2010 eruption^23^. It is now described by EASA (European Aviation Safety Agency) and used in the emergency plan EUR/NAT (EURopean and North ATlantic office) as the “High” contamination level.

Therefore, in order to test the sensitivity of concentration variations to partitioning values, distal ash cloud dispersion maps were simulated for 4 eruption scenarios (Supplementary Information Table S1) using MOCAGE-accident, the ash-cloud-dispersal model of VAAC Toulouse. This model is based upon the three-dimensional chemistry and transport model developed by Météo-France, and specifically adapted for the transport and diffusion of accidental release from the regional to the global scale. For this study, meteorological data were extracted from Météo-France operational database, including 20 pressure levels, from 1000 to 10 mb, with a time resolution of 1-hour and a horizontal resolution of 0.5°. MOCAGE-accident internal grid resolution is 0.5°. For each scenario, ash release was constant for the eruption phase duration and uniform along a vertical line rising from the vent to the maximum plume height. The particle size distribution in the distal cloud includes 6 grain size fractions between 0.1 and 100 µm, with 70 wt% of the particles smaller than 30 µm^36^. For modelling simplicity, we run the simulations using present-day meteorological data. For the Plinian case (see Supplementary Information Fig. S2 for Subplinian and Small/Moderate cases), we use an eruptive scenario based on the Kelut 2014 eruption (Supplementary Information, Table S1). We compare the ash cloud loading (i.e., integration of ash concentration along the vertical path; in kg/m^2^) simulated using the VAAC-default *ε* value (5%) with the Plinian partitioning coefficient (*ε*_*P*_ = 0.5%) derived from our model (Fig. 2). The ash cloud concentrations are drastically different, with maximum values of 1.7 × 10^−1^ and 1.6 × 10^−2^ kg/m^2^ for the VAAC-default and our eruption-style-dependant coefficients, respectively (Fig. 2a,b). This means that for such Plinian eruptions, VAAC operational simulations could overestimate by a factor of ∼10 the amount of very fine ash in the atmosphere. Consequently, this would overestimate the extent of the no-fly zone (delimited in Fig. 2 by the black dashed line) set by the European Commission beyond a threshold^37^ of 4 mg/m^3^ (Fig. 2). Patterns of the no-fly zones are drastically different, and the extent computed from the VAAC model is ∼6.5 times larger than the other one, which could have serious implications for air traffic regulation during an eruption.

Volcanic ash particles can be responsible for the formation of indirect aerosols and/or droplets, the ones potentially having short term effect on the climate^38–40^. However, the systematic overestimation of the fine ash amount injected in the atmosphere during large sustained eruptions raises questions about the actual impact of volcanic ash on radiative forcing Conversely, when no calibration is available from ground deposits, the proximal sedimentation can be underestimated by such models (or other tephra-deposition models), as collective settling mechanisms are still not well constrained. This raises the question of the actual impact (buildings damage, agriculture and water pollution, health and respiratory problems, etc.) of tephra fallout in the vicinity of volcanic areas, likely to be larger than expected.

### Satellite-based prediction model of *Q*_*s*_

**Figure 3:**
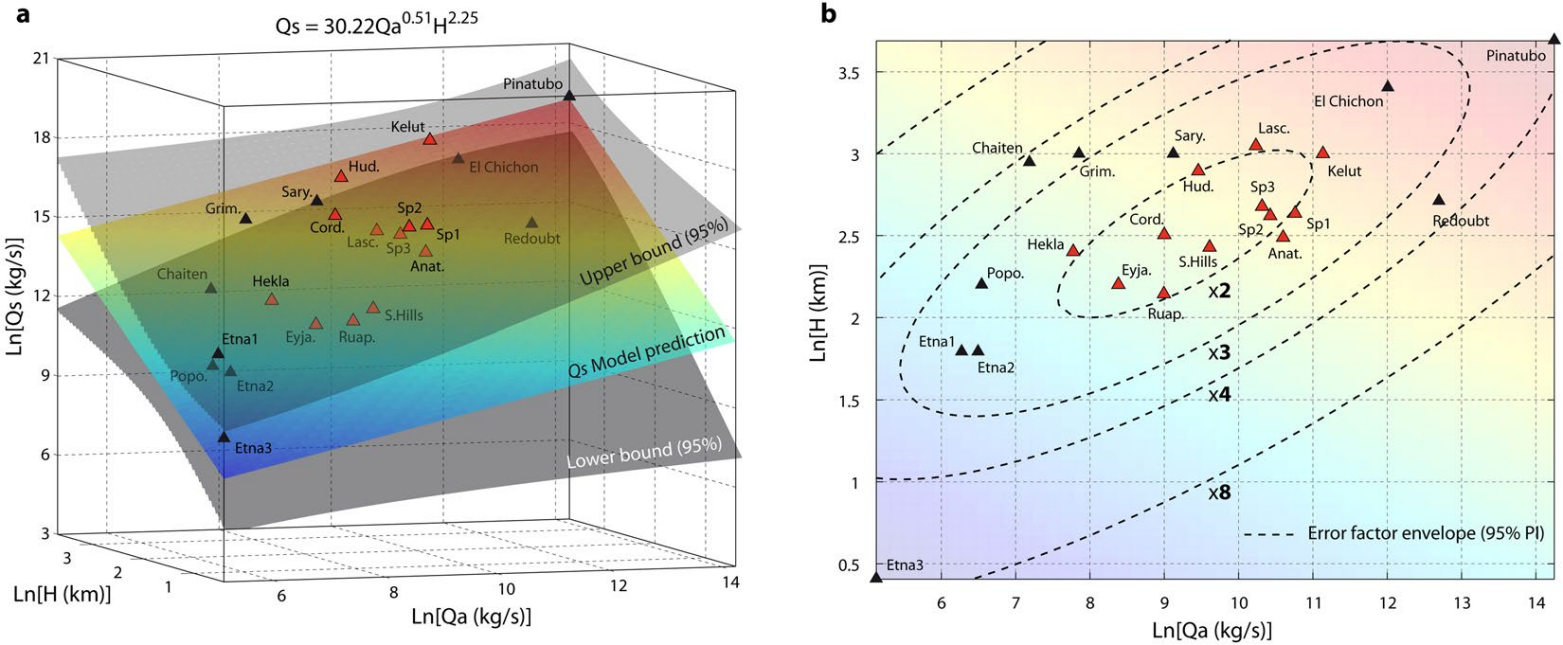
*Q*_*s*_ prediction model using model selection analysis. (**a**) Statistical relationship between *Q*_*s*_ derived from fallout deposits, *Q*_*a*_ derived from satellite-based measurements, and H (above the vent) derived from observations, in a three-dimensional natural logarithm space. The best *Q*_*s*_ prediction model is shown as the coloured plane and the related equation is given in natural scale following a power-law at the top of the plot. It was selected by the AICc (Corrected Akaike Information Criterion) which gives a robust evaluation of the goodness-of-the-fit for small datasets. The error factor and related RMSE are provided at a 95% prediction interval. See Supplementary Information Table 2 for all the goodness-of-the-fit evaluation criteria. (**b**) Error factor contour levels related to the MER estimation plotted on the two-dimensional plane H vs. *Q*_*a*_ in natural logarithm, and showing the anisotropy of the error distribution. Red triangles represent eruptions (12 over 22) for which the error factor value is ∼2 or less. Practically, this means that estimations of MER for future eruptions falling in this range of *Q*_*a*_ (∼1 × 10^3^ to 1 × 10^5^ kg/s) and H (∼7 to 21 km) have a 95% probability to fall within an error factor of 2 only.

The interdependence of *Q*_*a*_, *Q*_*s*_ and the eruption style leads us to develop statistical models for predicting *Q*_*s*_ using satellite measurements of *Q*_*a*_ with additional controlling parameters. A reliable assessment of *Q*_*s*_ is essential for estimating plume dynamics close to the source, and hence for delineating zones impacted by tephra fallout using tephra-deposition models^41^. However, direct measurements of *Q*_*s*_ remain impossible during the course of an eruption^42^. Thus, for rapid assessment of *Q*_*s*_, indirect methods have been developed using scaling laws based on relationships between measured plume height *H* and time-averaged *Q*_*s*_; these are referred to as empirical scaling laws^35,43^. This methodology currently represents the standard for real-time determination of *Q*_*s*_, although associated with uncertainties as large as a factor of 54 at a 95% confidence interval^35^. Data investigated here are small sized while the number of explanatory variables is relatively high. Therefore, we developed specifically a novel and robust statistical technique using a modified Akaike Information Criterion (AICc; see Methods) allowing the selection of the best regression mixture model for the eruptions in our database (all statistical indicators are summarized in Table 2). By combining *Q*_*s*_, *Q*_*a*_ and *H* in three-dimensional space (Fig. 3a), the best model selected follows a power-law in the form:

1 $${Q}_{s}=30.22{Q}_{a}^{0.51}{H}^{2.25}$$

**Table 2:** Summary of statistic results using model selection analysis.

Models	Coefficients		p-value	AICc	RMSE (95%)	Error factor
Qs = c_0_Qa^c1^	c0	89.211	5.93E-03***	22.49	3.27	26.31
	c1	1.004	2.13E-06***			
Qs = c_0_Qa^c1^H^c2^	c0	30.220	8.87E-03***	12.93	2.55	12.80
	c1	0.505	1.05E-02**			
	c2	2.249	1.31E-03***			
Qs = c_0_Qa^c(P1)^H^c(P3)^	c0	25.946	2.16E-02**	10.43	2.23	9.29
	c(P1_(lo)_)	0.721	5.65E-03***			
	c(P1_(hi)_)	0.622	2.49E-03***			
	c(P3_(cl)_)	1.950	1.80E-03***			
	c(P3_(op)_)	1.396	3.17E-02**			

p-values quality is illustrated using asterisk (***Excellent; **very good). The AICc stands for corrected Akaike Information Criterion, the RMSE is given as the natural logarithm of the Root Mean Square Error for a 95% prediction interval and the error factor is calculated as the exponential of the RMSE.

This relationship gives an AICc of 12.9 with excellent p-values (Table 2). The RMSE (Root Mean Square Error) yields an error factor of 12.8 at a 95% prediction interval. With an uncertainty four times lower than the empirical scaling laws^35^, this new satellite-derived model improves significantly the estimation of *Q*_*s*_ (Table 2). In particular, the error distribution is not uniform as shown in Fig. 3b from the projection of the 95% prediction interval envelope in the *H-Q*_*a*_ plane. This yields an error factor of ∼2, close to the data centre of mass that encloses 12 of the 22 eruptions of our dataset.

Then, we also collected 5 additional parameters (P_1_ to P_5_, Table 3) related to magmatic system properties and external processes (referred to as modalities), likely to control the amount of very fine ash produced and injected in the plume. Each modality has been coded on a Boolean basis (0/1) so that they can be statistically analysed. We then proceeded to the selection steps to discriminate between all the possible models with 7 different variables (*Q*_*a*_, *H*, *P*_1_ *to P*_5_), with modalities (*P*_1_, …, *P*_5_) being class parameters for *Q*_*a*_ and *H*. The modalities include the SiO_2_ (P_1_) and H_2_O (P_2_) contents of the magma, the open or closed character of the conduit (P_3_), the occurrence of phreatomagmatic activity (P_4_), and the formation of co-pyroclastic density current (co-PDC) plumes (P_5_). Using our selection model analysis, these modalities allow clustering of the 22 data samples in the 3D space defined by *Q*_*s*_, *Q*_*a*_ and *H*, and the identification of sub-models corresponding to different eruption scenarios (see the Methods section for details). We found that P_1_ and P_3_ are the parameters that best improve the fitness criterion, with a low AICc value of 10.4. This leads to a new sub-model yielding an error factor of 9.3 at a 95% prediction interval based on four different equations as follows:

2 $${Q}_{S}=25.95{Q}_{a}^{0.72}{H}^{1.95}\,\,{\rm{low}} \mbox{-} {{\rm{SiO}}}_{2}\,{\rm{and}}\,{\rm{closed}} \mbox{-} {\rm{conduit}}$$

3 $${Q}_{S}=25.95{Q}_{a}^{0.72}{H}^{1.4}\,\,{\rm{low}} \mbox{-} {{\rm{SiO}}}_{2}\,{\rm{and}}\,{\rm{open}} \mbox{-} {\rm{conduit}}$$

4 $${Q}_{S}=25.95{Q}_{a}^{0.62}{H}^{1.95}\,\,{\rm{high}} \mbox{-} {{\rm{SiO}}}_{2}\,{\rm{and}}\,{\rm{closed}} \mbox{-} {\rm{conduit}}$$

5 $${Q}_{S}=25.95{Q}_{a}^{0.62}{H}^{1.4}\,\,{\rm{high}} \mbox{-} {{\rm{SiO}}}_{2}\,{\rm{and}}\,{\rm{open}} \mbox{-} {\rm{conduit}}$$

**Table 3:** Explicative variables used in the statistical analyses for the 22 eruptions.

Volcano	Explosive Phase selected	P_1_ SiO_2_ content (wt%)	Coding	P_2_ Inclusion Depth (km)	H_2_O (wt%)	Coding	P_3_Open/Closed conduit	Coding	P_4_Phreato-magmatism	Coding	P_5_Co-PDC plumes	Coding
Pinatubo	Climactic phase on 15–16/06/1991	78^(49)^	1	8.5	6.5^(49,50)^	1	Closed^(51)^	0	No^(52)^	0	Yes^(53)^	1
Kelut	Full eruption 13/02/2014	56^(5)^	1	19	4.72^(54)^	0	Closed^(55)^	0	No^(55)^	0	Yes^(56)^	1
El Chichon (events B & C)	Phases B and C on 04/04/1982	55.9^(57)^	1	8	4^(58)^	1	Closed^(59)^	0	Yes^(59)^	1	Yes^(59)^	1
Hudson	Full eruption 12–15/08/1991	60–65^(60)^	1	4	3^(60)^	1	Closed^(10)^	0	Yes^(61)^	1	No^(10)^	0
Sarychev Peak	Subplinian events on 14–15/06/2009	54.4^(12)^	0	3.5	4^(12)^	1	Closed^(12)^	0	No^(12)^	0	Yes^(12)^	1
Cordón Caulle	Climactic phase on 4–5/06/2011	67–70^(62)^	1	3.75	2.7^(PC)^	1	Closed^(63)^	0	No^(63)^	0	Yes^(64)^	1
Grimsvotn	Subplinian phase on 22/05/2011	50^(65,66)^	0	15	0.7^(66)^	0	Closed^(66)^	0	Yes^(67)^	1	No^(66)^	0
Mt. Spurr (1)	Full eruption 16/09/1992	57^(68)^	1				Closed^(69)^	0	No^(70)^	0	No^(71)^	0
Mt. Spurr (2)	Full eruption 18/08/1992	57^(68)^	1				Closed^(69)^	0	No^(70)^	0	No^(71)^	0
Redoubt (events 1 to 5)	Explosive events 1 to 5 on 22–23/03/2009	57–62^(72)^	1	5	4.9^(73)^	1	Open^(22)^	1	No^(22)^	0	No^(22)^	0
Mt. Spurr (3)	Full eruption 27/06/1992	57^(68)^	1				Closed^(69)^	0	No^(70)^	0	No^(71)^	0
Lascar	Full eruption 04/1993	57–61^(74)^	1	4.5	5^(75)^	1	Open^(74)^	1	No^(74)^	0	Yes^(76)^	1
Anatahan	Explosive phases on 10–11/05/2003	60–61^(77)^	1	1	3.4^(78)^	1	Closed^(78)^	0	Yes^(78)^	1	No^(79)^	0
Chaiten	Full eruption: 2–8/05/2008	76^(80)^	1	5	2.3^(80)^	0	Closed^(80)^	0	No^(80)^	0	No^(28)^	0
Hekla	Phase I + 8 hrs phase II on 26/02/2000	55.5^(81)^	0	9	2.5^(82)^	0	Closed^(81)^	0	Yes^(29)^	1	Yes^(81)^	1
Soufrière Hills	Full eruption: 26/09/1997	57–61^(83)^	1	4.5	4.91^(84)^	1	Open^(85)^	1	No^(85)^	0	Yes^(32)^	1
Ruapehu	Full eruption: 17/06/1996	57.5–62^(86)^	1	1.5	1.9^(86)^	1	closed^(87)^	0	No^(87)^	0	No^(87)^	0
Eyjafjallajökull	Phase I/III on 14–19/04 & 05–18/05/2010	56.6–61.4^(88)^	1	5	1.8^(89)^	0	Closed^(90)^	0	Yes^(91)^	1	No^(92)^	0
Etna (1)	Full eruption: 28/10/2002	47^(93)^	0	4	3.4^(93)^	1	Open^(94)^	1	No^(94)^	0	No^(94)^	0
Popocatepetl	Climactic events on 10/03/1996	59.8–63.1^(95)^	1	10	3.2^(96)^	0	Open^(44)^	1	Yes^(44)^	1	No^(44)^	0
Etna (2)	Full eruption: 27/10/2002	47^(93)^	0	4	3.4^(93)^	1	Open^(94)^	1	No^(94)^	0	No^(94)^	0
Etna (3)	Full eruption: 24/11/2006	48^(93)^	0	4	3^(93)^	1	Open^(94)^	1	No^(94)^	0	No^(94)^	0

The five explicative variables considered here (SiO_2_ (*P*_*1*_) and H_2_0 (*P*_*2*_) magma content, open or closed character of the conduit (*P*_3_), occurrence of phreatomagmatism (*P*_4_), and formation of co-PDC plumes (*P*_5_)) are coded as Boolean data type (0/1) to be used in the model selection analysis. The threshold between low and high SiO_2_ content is set at 56 wt% for *P*_1_. The threshold between low and high water saturation for *P*_2_ is set considering the inclusion entrapment depth. Missing data for *P*_2_ are compensated by the model.

The magma SiO_2_ content (P_1_), often associated with the magma viscosity is a critical parameter controlling the pressurization state of the shallow magmatic system provided sufficient gas is available. The parameter (P_3_) related to the open/closed character of the conduit goes in the same direction. Indeed, closed systems usually designate volcanic conduits or vents sealed by cooled lava acting as an impermeable plug preventing from easy gas exhaust, and hence allowing a pressure increase in the shallow magmatic system. Exclusion of P_2_ is unexpected, as the gas usually controls the MER at the source vent. This can be explained by the difficulty of comparing H_2_O content measurements made with different techniques. Exclusion of P_4_ is also interesting. Indeed, the phreatomagmatism is a mechanism involving external water and is frequently observed during recent subglacial Icelandic eruptions^44^. In one hand, magma-water interaction can enhance the explosivity hence the formation of very fine ash. On the other hand, water-rich eruptive column is likely to cause premature deposition of ash through wet aggregation and hydrometeor formation^14,29^. However, no significant influence of the phreatomagmatism could be demonstrated by the our statistical analysis. Significant amount of very fine ash can be produced by PDC as for MSH1980 Plinian eruption, therefore the contribution to airborne ash by co-PDC plumes needed to be tested. However, the variability of co-PDC plumes (P_5_) dispersion mechanisms^45^ associated with the difficulty to assess quantitatively their amplitude is likely to explain their exclusion. The power-law coefficients are related to the modalities (*P*_*n*_) and show a strongly non-linear behaviour with power values of 0.72 and 0.62 on *Q*_*a*_ for low and high-SiO_2_, respectively, and power values of 1.95 and 1.4 on *H* for closed and open-conduit respectively. The constant (c_0_ = 25.95) is inherent to the general model structure and is not dependent on the explanatory variable *Q*_*a*_ and *H*, nor on the modalities P_1_ and P_3_.

**Figure 4:**
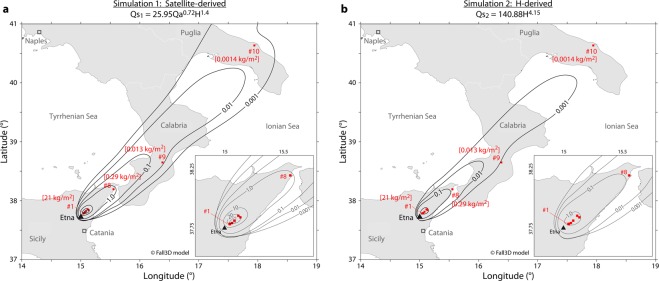
Simulations of the tephra fallout deposit from the 23^rd^ February 2013 Etna eruption. The simulations are generated by the FALL3D tephra-transport deposition model with distinctive *Q*_*s*_ as input. The simulated tephra fallout deposits are displayed as isomass contour levels (black lines) that represent the “computed tephra load” on the ground in kg/m^2^. The “measured tephra load” on the ground is indicated in red squares at individual locations of field sampling (red squares and numbers, see Supplementary Information Table 3 for details on sampling locations). (**a**) Simulation 1 uses an input *Q*_*s*1_ estimated with our satellite-derived statistical model (see equation at the top of the map for low SiO_2_ content and open system). The simulated deposit is in very good agreement with the “measured tephra load” at locations #1, 8, 9 and 10 for instance. (**b**) Simulation 2 uses an input *Q*_*s*2_ estimated with the empirical scaling law^35^ (see equation at the top of the map). The simulated deposit has a much smaller extent than in simulation 1, with “computed tephra loads” departing significantly from the “measured tephra loads”. The total erupted mass (TEM) according to these simulations yields values of 1.09 × 10^10^ and 6.58 × 10^8^ kg for simulation 1 and 2, respectively. The reference TEM value^47^ for this fallout deposit is 4.9 × 10^9^ kg which means that the Satellite-derived statistical model overestimates the TEM by a factor of ∼2.2, while the empirical scaling law underestimates the TEM by a factor ∼7.4.

Equations 2 to 5 offer a new tool for accurate, near-real-time estimation of *Q*_*s*_ during an eruption, provided that *Q*_*a*_ and *H* can be estimated. In order to validate our approach, we simulated the 23 February 2013 eruption of Mount Etna (Sicily) using two different *Q*_*s*_ inputs. The goal of this work is to test the ability of each parameterization (*Q*_*s1*_ and *Q*_*s2*_) to reproduce the observed tephra fallout deposits. Simulations have been carried out using Fall3D; which is a tephra-transport and deposition model, and now represent a standard used at INGV (Italy), VAACs of Buenos Aires (Argentina) and Darwin (Australia). Thus, Fall3D is a perfect candidate for this analysis; a full description of its characteristics can be found in the litterature^41,46^. In one hand, *Q*_*s1*_ was estimated from our satellite-derived statistical model using the parameterization for low-SiO_2_ content and open conduit (Eq. 3), and used as input parameter in simulation 1 (Fig. 4a). On the other hand, *Q*_*s2*_ was calculated from the standard empirical scaling law^35^ (currently used operationally by the London and Toulouse VAAC), and used as input parameter in simulation 2 (Fig. 4b). Simulations were run between 00:00 (all times are in UTC) on the 23 and 24:00 on the 28 February 2013, within a 445 by 445 km grid domain using meteorological fields (from ECMWF data). They include 37 pressure levels with a time resolution of 6 hours and a horizontal resolution of 0.75°. The FALL3D internal grid resolution was 4 by 4 km, obtained by interpolating linearly the meteorological data. The three main Eruption Source Parameters (ESP) required by FALL3D at the input of the model are the plume column height, the Total Grain-Size Distribution and *Q*_*s*_. Then, the ability of each model to reproduce the observed tephra fallout deposits is assessed using field measurements^47^ of tephra loading at 10 locations carried out after the 23 February 2013 eruption of Mount Etna (Fig. 4; Supplementary Information Table S2, Table S3 and Fig. S3). The two simulations are strikingly different. The first one (Fig. 4a) provides a faithful reconstruction of the deposits as shown by the 5 isomass contours (set at 10, 1, 0.1, 0.01, and 0.001 kg/m^2^) correctly enclosing the sampling points #1 (21 kg/m^2^), #8 (0.29 kg/m^2^), #9 (0.013 kg/m^2^), and #10 (0.0014 kg/m^2^). On the contrary, the simulation 2 (Fig. 4b) using the empirical H-derived scaling law fails at reproducing the actual deposits and significantly underestimates the amount of tephra deposited on the ground. This is clearly shown by the restricted extent of the computed isomass contours, and is the direct consequence of the underestimation of *Q*_*s*_. These results illustrate the robustness of our model and highlight the importance of including satellite-derived estimates of *Q*_*a*_ for reliable estimations of *Q*_*s*_.

## Conclusion

Volcanic very fine ash clouds can travel great distances and contaminate the atmosphere for long periods of time, disrupting air traffic as demonstrated during recent eruptions. However, the proportion of very fine ash distally transported in the atmosphere, and related proximal settling processes, are difficult to assess. Yet, for the past two decades, several operational meteorological agencies (VAACs) have used an unrealistic default value of ε = 5% as input for forecast models of atmospheric ash cloud concentration. Here, from the combination of field and satellite data, we provide first-time quantitative assessment of the source-to-atmosphere partitioning (ε) of very fine ash from 22 eruptions. We also developed a robust and novel statistical model for predicting the source mass eruption rate (*Q*_*s*_) with an unprecedentedly low level of uncertainty. The main findings are summarized below:

i. The fraction of very fine ash (i.e., which survive proximal settling) varies by ∼2 orders of magnitudes (0.1 > *ε* > 6.9%) with respect to the MER. This partitioning is not arbitrary as *ε* decreases with increasing MER, with respect to eruption styles.
ii. Large plumes from Plinian eruptions are much less efficient (up to 50 times lower) at transporting very fine ash through the atmosphere than previously anticipated.
iii. We explain this behaviour by the existence of collective particle settling mechanisms occurring in ash-rich plumes, which enhance early and en masse fallout of very fine ash.
iv. We suggest that proximal sedimentation during powerful eruptions is controlled by the concentration of fine ash regardless of the grain size.
v. We thus propose a style-derived parameterization of ε (ε_P_ = 0.5%; ε_SP_ = 0.8%; ε_S/M_ = 3.2%) to be used into VAAC ash-cloud-dispersal models for operational applications.
vi. We provide a novel and robust statistical model for the estimation of the source Mass Eruption Rate (*Q*_*s*_), with an unprecedented reduction of uncertainties from an error of a factor 54 (previous work used by some VAACs) to a factor 9.3 at a 95% prediction interval.

The fact that very fine ash from Plinian eruptions are not efficiently transported in the atmosphere and experience early sedimentation has major implications for risk management. On the ground, tephra fallouts can be more severe than predicted by current tephra-deposition models, having a detrimental effect on water infrastructure, buildings or agriculture. In the atmosphere, the concentration of far-travelled ash clouds can be much lower than predicted by current ash-cloud-dispersal models, hence having important impact for crisis management related to air traffic safety. We propose incorporating our eruption-style-dependant partitioning coefficients into VAAC ash-cloud-dispersal models, as well as the use of the equations (2–5) of our statistical model into tephra-deposition models. For this purpose, we provide (Supplementary Information, Table S4) operational parameters to be used in real-time for three standard eruptive scenarios (i.e., Plinian, Subplinian, and Small/Moderate). For each scenario, these parameters include the Total Grain Size Distribution^48^ (TGSD), the total ash fraction with diameter <64 µm, the distal very fine ash fraction (ε_P_, ε_SP_, ε_S/M_), and equations of *Q*_*s*_ for the estimation of the source mass eruption rate.

## Methods

### Statistical model

Data investigated here are small sized while the number of explanatory variables is relatively high. A classical solution consists in regularizing parameters estimation by introducing a penalty term into the maximum likelihood estimation problem. For instance, Ridge or Lasso regressions are based on this principle and have been introduced for variable grouping or to reduce the residuals variance^49,50^. However, each one is either specialized in the selection (grouping of variables) or in the reduction of quadratic errors^51–53^. Consequently, and in adequacy with our context, we propose to introduce a new penalty term that will allow: (i) grouping explanatory variables to determine the relevant number of predictors (ii) improving the estimation of the parameters assigned to each class and (iii) taking into account the small size of observed data. To avoid making any *a-priori*, the methodology has been at first, set in the general context of a Gaussian regression mixture models but it turned out by investigating the data set that only one Gaussian regression model is selected via our procedure. Therefore, we only present our methodology in this context, which moreover allows physical interpretations of the involved parameters. Indeed, consider (*y*_1_, …, *y*_*n*_)^*t*^ a sample observed from the interest variable *Y* (Mass Eruption Rate, *Q*_*s*_) and let (*x*^*t*^, …, *x*^*t*^)^*t*^ be a matrix of explanatory variables *X* (*Q*_*a*_, *H*, *P*_1_…, *P*_5_); *x*_*i*_ are vectors of ℜ^*P*^. The estimation problem reduces to maximize the following penalized log-likelihood function:

$$l(\beta ,\sigma ,Y,X)=\sum _{i=1}^{n}\,log\,f(y,{x}^{t}\beta ,\sigma )-\,{\boldsymbol{Pe}}(\beta )$$

where *f* (*y*, *x*^*t*^*β*, *σ*) is a Gaussian probability density with mean *x*^*t*^*β* and variance *σ*^2^. The penalty term **Pe** (.) is given by $Pe(\beta )=\alpha \sum _{j=1}^{p}\,|\beta |+(1-\alpha )\sum _{j=2}^{p}\sum _{l=1}^{j-1}\,|{\beta }_{j}-{\beta }_{l}|$. The quantity 0 < α < 1 is a tuning parameter whose optimal choice makes a balance between the error of the model and the numbers of predictors used in it. This procedure is confirmed and emphasized by using model’s selection Criterion. The best known is the Akaike Information Criterion (AIC). It was designed as an asymptotically unbiased estimator of the Kullback divergence between the true model (that actually generated the data) and a statistical approximation of it. The measure of separation between the generating and a candidate model that we use is given by the Kullback’s symmetric divergence^54^. If we denote Φ = (*β*, *σ*) and Φ_0_ = (*β*_0_, *σ*_0_) this divergence is defined by:

$$J({{\rm{\Phi }}}_{0},{\rm{\Phi }})=\{d({{\rm{\Phi }}}_{0},{\rm{\Phi }})-d({{\rm{\Phi }}}_{0},{{\rm{\Phi }}}_{0})\}+\{d({\rm{\Phi }},{{\rm{\Phi }}}_{0})-d({\rm{\Phi }},{\rm{\Phi }})\}$$

where *d*(Φ_0_, Φ) = E_Φ0_ {−2 log *f* (Y |Φ)} is the Kullback-Leibler divergence and E_Φ0_ denotes the expectation with respect to *f* (*Y|*Φ_0_). Since *d*(Φ_0_, Φ_0_) does not depend on Φ we use:

$$K({{\rm{\Phi }}}_{0},{\rm{\Phi }})=d({{\rm{\Phi }}}_{0},{\rm{\Phi }})+\{d({\rm{\Phi }},{{\rm{\Phi }}}_{0})-d({\rm{\Phi }},{\rm{\Phi }})\}$$

For large sample data and inspired by^55^, one may prove that the criteria defined by:

$$AIC=nlog\,{\hat{\sigma }}^{2}+2(p+1)$$

is asymptotically unbiased estimator of E_Φ0_ (d(Φ_0_, $\hat{{\rm{\Phi }}}$)). In the case of small samples, we may prove that the criteria defined by:

$$AICc=nlog\,{\hat{\sigma }}^{2}+2\frac{n(p+1)}{n-p-2}$$

is unbiased estimator of E_Φ0_ (d(Φ_0_, $\hat{{\rm{\Phi }}}$)) and still satisfy the same asymptotic properties than the AIC. We say that a model is selected through the AIC_c_ if it has the lowest AIC_c_ in the family of chosen models.

Let us first observe that due to the range of the observations value, it is natural to consider log(*y*) as our new observations. The Gaussianity and independence of the observations will be asserted once the selection procedure is performed. Secondly, due to the very small number of observations, with respect to the number of covariables and using parameter estimations in a complete model, we then proceed to the selection steps to discriminate between all the possible models with 7 different variables (*Q*_*a*_, *H*, *P*_1_, …, *P*_5_). To this end, let us remark that *Q*_*a*_ and *H* are physical parameters (directly related to *Q*_*s*_), while other parameters (here named *P*_1_ to *P*_5_) are related to magmatic system properties and external processes likely to impact *Q*_*s*_. We thus choose (*P*_1_, …, *P*_5_) to be class parameters for *Q*_*a*_ and *H*, which is natural from a physical interpretation of the different volcanoes and meteorological conditions. Namely, we test models depending on the modality of the parameters leading to the complete model with unknown parameters generically called *β*_*·*_

$${\rm{l}}{\rm{o}}{\rm{g}}\,{y}^{k}={\beta }_{0}+\sum _{i=1}^{7}\,{\beta }_{i}{P}_{i}^{k}+\sum _{j=1}^{2}\sum _{i=3}^{7}\,{\beta }_{j,{P}_{i}^{k}}{P}_{j}^{k}+\sigma {\xi }^{k}$$

where (*ξ*^*k*^) are independent standardized Gaussian variable. The selection via *AIC*_*c*_ criterion is then performed.

## Supplementary information

Supplementary Information

## Data Availability

All data generated or analysed during this study are included in this published article (and its Supplementary Information files).
