# Author Correction: Low efficiency of large volcanic eruptions in transporting very fine ash into the atmosphere

**DOI:** 10.1038/s41598-019-42489-z

**Published:** 2019-04-10

**Authors:** Mathieu Gouhier, Julia Eychenne, Nourddine Azzaoui, Arnaud Guillin, Mathieu Deslandes, Matthieu Poret, Antonio Costa, Philippe Husson

**Affiliations:** 1 Université Clermont Auvergne, CNRS, IRD, OPGC, Laboratoire Magmas et Volcans, F-63000 Clermont-Ferrand, France; 2 Laboratoire de Mathématiques Blaise Pascal, UMR 6620 CNRS & UCA, Aubière, France; 3 VAAC Toulouse, Météo France, Toulouse, France; 4 Istituto Nazionale di Geofisica e Vulcanologia, Sezione di Bologna, Bologna, Italy

Correction to: *Scientific Reports* 10.1038/s41598-019-38595-7, published online 05 February 2019

The Acknowledgements section in this Article is incomplete.

“J.E. was funded by the Research Institute for Development (IRD). We are particularly grateful to Timothy H. Druitt for constructive scientific discussions. We warmly thank A. Folch and one anonymous reviewer for constructive comments.”

should read:

“J.E. was funded by the Research Institute for Development (IRD). We are particularly grateful to Timothy H. Druitt for constructive scientific discussions. We warmly thank A. Folch and one anonymous reviewer for constructive comments. We thank the European project EUROVOLC (grant number 731070), A.C. also acknowledges the Italian MIUR project Premiale Ash-RESILIENCE.”

