# Supplementary material for: Low efficiency of large volcanic eruptions in transporting very fine ash into the atmosphere
Source: Sci Rep. 2019 Feb 5;9:1449. doi: 10.1038/s41598-019-38595-7 (PMC6363761; doi:10.1038/s41598-019-38595-7)
Supplement: Supplementary file 1 — Supplementary Information [file 41598_2019_38595_MOESM1_ESM.pdf]

Supplementary information: Low efficiency of large volcanic eruptions in transporting very fine ash into the atmosphere

Mathieu Gouhier[1*], Julia Eychenne[1], Nourddine Azzaoui[2], Arnaud Guillin[2], Mathieu Deslandes[3], Matthieu Poret[4], Antonio Costa[4], Philippe Husson[3]

[1]Université Clermont Auvergne, CNRS, IRD, OPGC, Laboratoire Magmas et Volcans, F-63000 Clermont-Ferrand, France
[2]Laboratoire Mathématique, Université Clermont Auvergne, CNRS/IRD, OPGC, Clermont Ferrand;
[3]VAAC Toulouse, Météo France, Toulouse, France;
[4]Istituto Nazionale di Geofisica e Vulcanologia, Sezione di Bologna, Bologna, Italy;

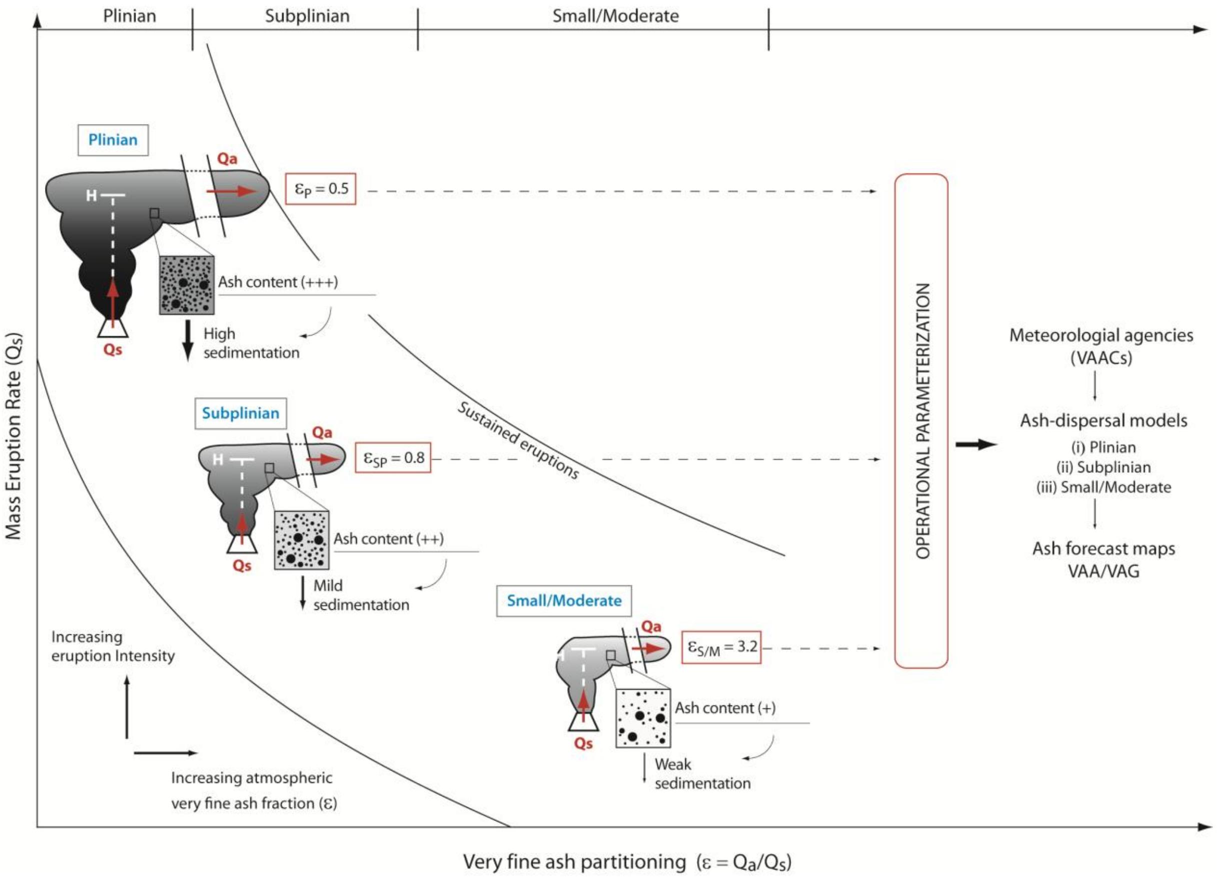

Supplementary Information Figure S1. Summary of the main findings. We show that the partitioning (ε) between the amount of tephra injected into the volcanic plume and the fine ash fraction that survive proximal sedimentation forming distal ash clouds is highly variable. During sustained eruptions (Plinian, Subplinian and Small/Moderate styles) this partitioning inversely scales with the eruption intensity (Q_s). Actually, the more the plume is concentrated in fine ash, the lower the proportion of fine ash injected into the atmosphere. This is due to the efficiency of collective particle settling in fine ash-rich plumes (such as Plinian and Subplinian) which enhance early and en masse fallout of fine ash, hence leading to very low ε values. We thus design a style-related operational parameterization using our partitioning coefficients (ε=0.5% for Plinian, ε=0.8% for Subplinian, and ε=3.2% for Small/Moderate) to be implemented as input parameters for ash-cloud-dispersal models run by the 9 VAACs (Volcanic Ash Advisory Centres) worldwide for the production of ash forecast maps.

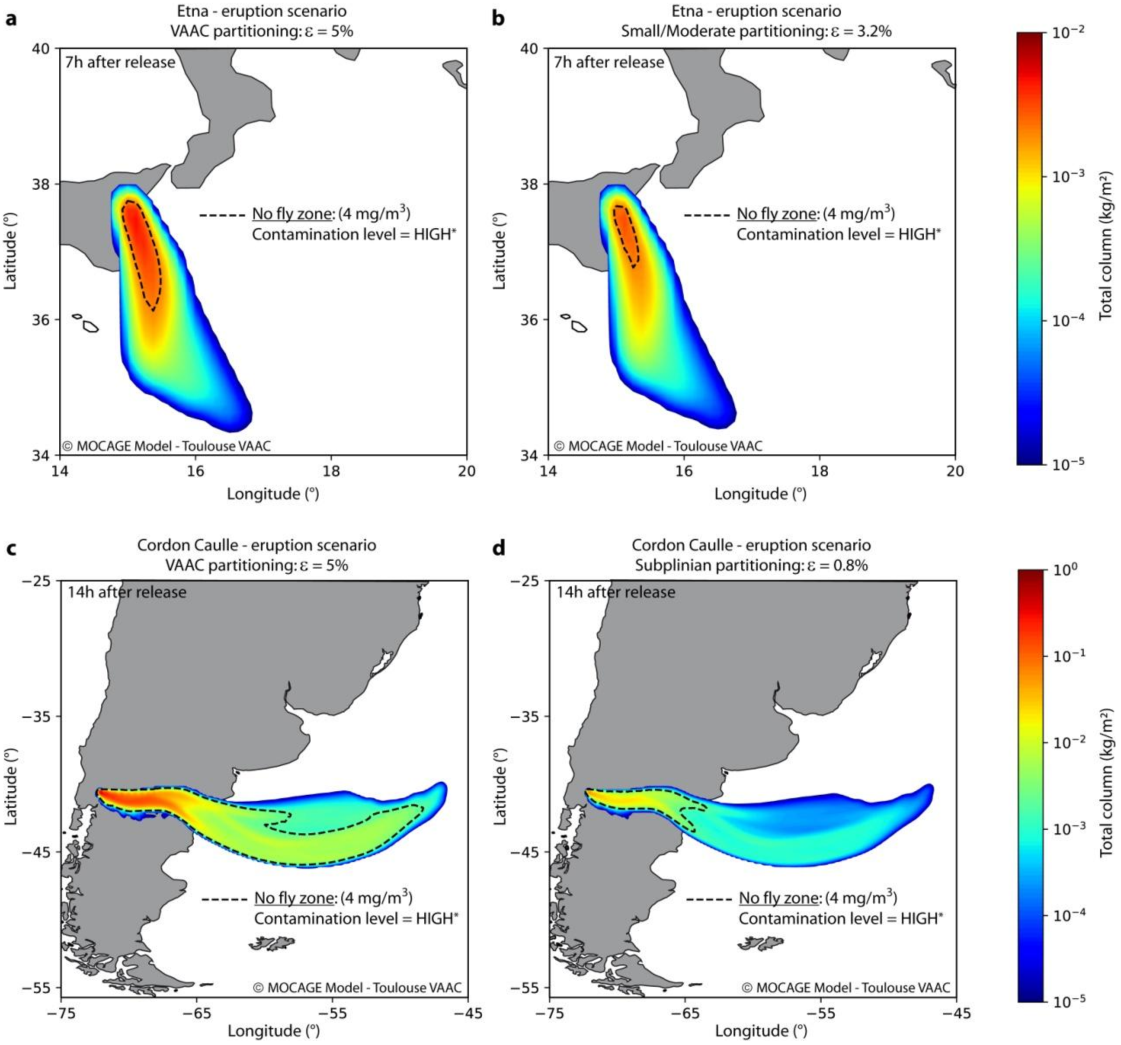

Supplementary Information Figure S2. Atmospheric ash concentration simulations. The four simulations are produced by the volcanic ash dispersion model MOCAGE of the Toulouse VAAC based on two eruptive scenarios, using different partitioning coefficients and present-day meteorological data. **a**, Simulation of ash dispersion in the atmosphere at Etna volcano 7 hours after a Small/Moderate eruption based on the 27 October, 2002 event (Supplementary Information Table 2), using the VAAC-default ε value of 5%. **b**, Same simulation conditions and scenario but using the Small/Moderate ε value established in this study at 3.2%. The extent of the No-Fly zone (4 mg/m^3 for an ash cloud 500-m thick*) is overestimated by the VAAC-default ε value. **c**, Simulation of ash dispersion in the atmosphere at Cordón Caulle volcano 14 hours after a Subplinian eruption based on the 4 June, 2011 event (Supplementary Information Table 2), using the VAAC-default ε value of 5%. **d**, Same simulation conditions and scenario but using the Subplinian ε value established in this study at 0.8%. The extent of the No-Fly zone (4 mg/m^3 for an ash cloud 500-m thick*) is greatly overestimated by the VAAC-default ε value. *The threshold at 4 mg/m^3 was first established by European commission after the Eyjafjallajökull 2010 eruption. It is now described by EASA (European Aviation Safety Agency) and used in the emergency plan EUR/NAT (EURopean and North ATlantic office) as the "High" contamination level.

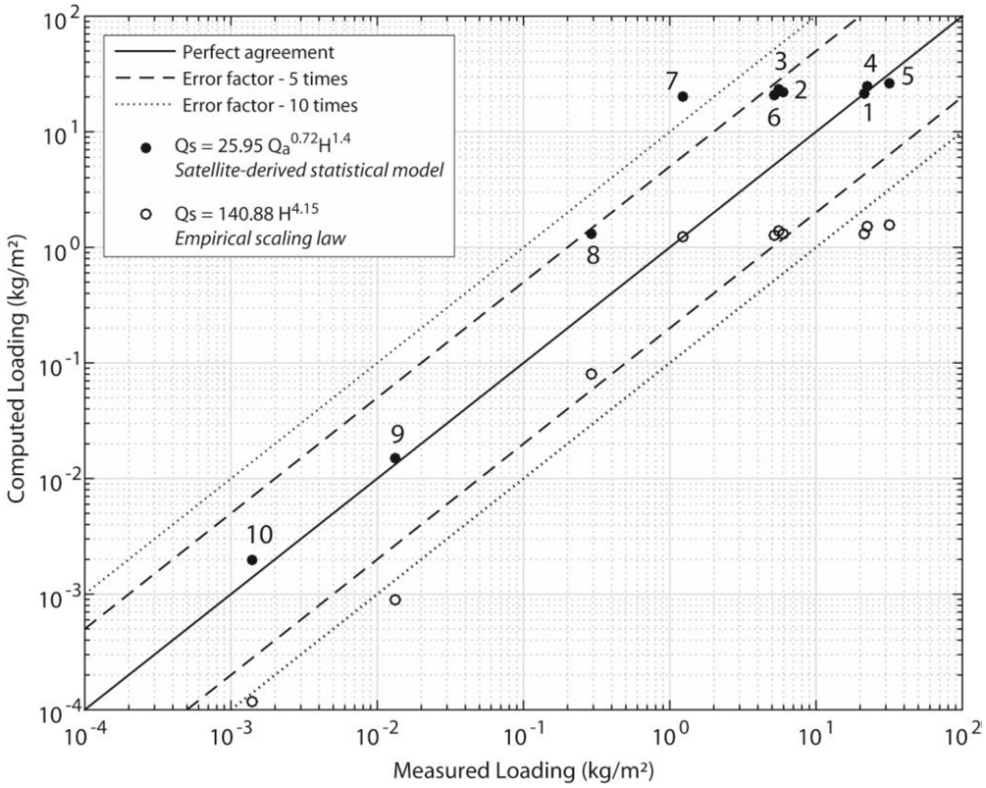

Supplementary Information Figure S3. Evaluation of the quality of the tephra deposition simulations of the 23rd February 2013 Etna eruption. The loading of tephra on the ground (x-axis) was measured at individual locations in the field, while the computed loading of tephra deposited on the ground (y-axis) at the same locations was generated by the FALL3D tephra-deposition model. All values and locations are listed in Supplementary Information Table 3. The filled circles correspond to the results of simulation 1 (Fig. 4a of the main text) using an input Q_s estimated with our satellite-derived statistical model (see equation in legend for low SiO_2 content and open system). In this case, all values fall within an error envelope of a factor of 5, except at location #7. At locations #1, 4, 5, 9 and 10 there is a perfect agreement with measured loading. The open circles correspond to the results of simulation 2 (Figure 4b of the main text) using an input Q_s estimated with the standard empirical scaling law (see equation in legend). Simulation 2 shows a larger discrepancy with the measured loading at location #1, 4, 5, 9 and 10, with an error factor larger than one order of magnitude.

	Kelut	Cordón Caulle	Etna
Eruptive style	Plinian	Subplinian	Small/Moderate
Eruption duration (hours)	3	24	10
Plume height (km)	20	13.7	3.65
Qs (kg/s)	6.02E+07	7.00E+06	2.42E+04
VAAC partitioning (ε)	5	5	5
Eruptive-style partitioning (ε)	0.45	0.8	2.23
TGSD (µm)	0.1-100*	0.1-100*	0.1-100*
Column mass distribution	Uniform	Uniform	Uniform
Meteo data resolution	0.5°x0.5°	0.5°x0.5°	0.5°x0.5°
Wind field time (T_0)	20170620 1800 UTC	20170620 1900 UTC	20170621 0100 UTC

Supplementary Information Table S1. Parameters of 3 eruptive scenarios for MOCAGE simulations (Toulouse VAAC). The 3 scenarios cover each eruptive style for sustained eruptions (Plinian, Subplinian and Small/Moderate) with related parameters used at the input of the MOCAGE model run by Toulouse VAAC. For each scenario, two simulations are computed using alternatively the VAAC operational partitioning coefficient (5%) and our modified partitioning coefficient depending of eruptive styles. *The Total Grain Size Distribution (TGSD) ranges from 0.1-100 µm and is split into 6 classes with 70% of the mass fraction within the 10-30 µm size range[48].

Id	Sampling Location	Distance (km)	Measured Load (kg/m2)	Measured Grain Size distribution mode (Φ)	Computed load Simulation 1	Computed load Simulation 2
1	Baracca	5.35	2.10E+01	-3.5	2.20E+01	1.30E+00
2	Casetta	5.45	5.90E+00	-4	2.20E+01	1.30E+00
3	Bivio-007	5.79	5.50E+00	-4	2.30E+01	1.40E+00
4	Forestale	7.32	2.20E+01	-3.5	2.50E+01	1.50E+00
5	Chalet	10.21	3.20E+01	-2.5	2.60E+01	1.60E+00
6	Castiglione	15.52	5.20E+00	-1.5	2.10E+01	1.30E+00
7	Linguaglossa	15.6	1.20E+00	-3	2.00E+01	1.20E+00
8	Messina	69.55	2.90E-01	1	1.30E+00	7.90E-02
9	Cardinale	157.17	1.30E-02	2	1.50E-02	9.00E-04
10	Brindisi	408.91	1.40E-03	3	2.00E-03	1.20E-04

Supplementary Information Table S2. Field data and results of the tephra deposition simulations of the 23 February Etna eruption. Simulations run with the volcanic tephra-deposition model FALL3D, using an input Q_s estimated with our satellite-derived statistical model (simulation 1, See Figure 4a in the main text and Supplementary Information Fig. 4), and an input Q_s estimated with the standard empirical scaling law (simulation 2, See Figure 4b in the main text and Supplementary Information Fig. 4).

Qs (kg/s)		H (km a.v.)	TTest	Corr.	Bias	*RMSE₁*	*RMSE₂*	*RMSE₃*	*K*	*k*	TEM (kg)
Satellite-derived stat. model	2.75×10^{6}	5.5	0.6	1.0	0.1	0.37	5.33	1.77	0.41	2.48	1.09×10^{10}
Empirical scaling law	1.66×10^{5}	5.5	0.2	1.0	-0.2	0.45	0.81	0.73	6.85	2.48	6.58×10^{8}

Supplementary Information Table S3. Statistical metrics evaluating the goodness-of-the-fit of the tephra deposition simulations of the 23rd February Etna eruption. *Corr* is the correlation coefficient between the observed and the simulated tephra loading values (Obs and Sim, respectively), expressed as $Corr(Obs, Sim) = \frac{Cov(Obs,Sim)}{\sigma_{Obs}\sigma_{Sim}}$, where, Cov is the covariance coefficient and σ is the standard deviation. *Bias* is expressed as $Bias = \frac{\sum_i Sim_i - Obs_i}{\sum_i Obs_i}$, where i represents the ith individual tephra sample. *TTest* is calculated as $TTest = \frac{\overline{Obs}-\overline{Sim}}{\sqrt{\frac{\sigma_{Obs}^2+\sigma_{Sim}^2}{N}}}$, where $\overline{Obs}$ and $\overline{Sim}$ are the means of the observed and simulated tephra loadings, respectively, and N is the number of samples. The TTest is a probabilistic coefficient that indicates how close the simulated and observed tephra loading values are. The three RMSEs are calculated using different hypotheses regarding the uncertainty on the ash loading values. $RMSE_1$ is calculated assuming that all the ash loading measurements have an equal uncertainty. $RMSE_2$ and $RMSE_3$ are calculated assuming a linear and uniform distribution, respectively, of the uncertainty on the ash loading measurements.

		Eruptive scenarios		
		Plinian	Subplinian	Small/Moderate
	phi [-log2(D in mm)]	wt.%	wt.%	wt.%
Total Grain Size Distribution (TGSD)**	-6	8.5E-01	2.2E-01	2.4E-04
	-5	1.8E+00	9.4E-01	1.7E-02
	-4	3.0E+00	2.9E+00	4.4E-01
	-3	4.4E+00	6.3E+00	4.4E+00
	-2	5.4E+00	9.8E+00	1.7E+01
	-1	5.8E+00	1.1E+01	2.6E+01
	0	6.1E+00	1.1E+01	1.7E+01
	1	8.3E+00	1.1E+01	9.9E+00
	2	1.4E+01	1.4E+01	1.0E+01
	3	1.9E+01	1.5E+01	8.7E+00
	4	1.7E+01	1.1E+01	4.8E+00
	5	1.0E+01	4.9E+00	1.7E+00
	6	3.7E+00	1.4E+00	3.7E-01
	7	8.6E-01	2.5E-01	5.0E-02
	8	1.3E-01	2.9E-02	4.2E-03
	9	1.2E-02	2.0E-03	2.2E-04
	10	7.3E-04	8.9E-05	7.5E-06
	11	3.6E-05	2.4E-06	1.6E-07
	12	2.1E-06	4.2E-08	2.0E-09
Total ash fraction	d<63µm (%)	31.6	17.4	6.9
Distal ash fraction	ε (%)	0.5	0.8	3.2
MER (Qs, kg/s)	closed-conduit	$25{,}95Qa^{0{,}62^*}H^{1{,}95}$	$25{,}95Qa^{0{,}62^*}H^{1{,}95}$	$25{,}95Qa^{0{,}72^*}H^{1{,}95}$
	open-conduit	$25{,}95Qa^{0{,}62^*}H^{1{,}4}$	$25{,}95Qa^{0{,}62^*}H^{1{,}4}$	$25{,}95Qa^{0{,}72^*}H^{1{,}4}$

Supplementary Information Table S4. Operational parameters for 3 standard eruptive scenarios.
*0,62 is for High-SiO_2 content and 0,72 is for low-SiO_2 content of the magma
** TGSD calculated[97] using magma viscosity (Log_{10} Pa.s) of 3,4 and 5 with plume height (km) of 5,15, and 25 for Small/Moderate, Subplinian, and Plinian eruptive scenarios, respectively.

Supplementary references

1 Holasek, R. E., Self, S. & Woods, A. W. Satellite observations and interpretation of the 1991 Mount Pinatubo eruption plumes. Journal of Geophysical Research: Solid Earth 101, 27635-27655, doi:10.1029/96JB01179 (1996).

2 Wiesner, M. G., Wetzel, A., Catane, S. G., Listanco, E. L. & Mirabueno, H. T. Grain size, areal thickness distribution and controls on sedimentation of the 1991 Mount Pinatubo tephra layer in the South China Sea. Bulletin of Volcanology 66, 226-242, doi:10.1007/s00445-003-0306-x (2004).

3 Guo, S., Rose, W. I., Bluth, G. J. S. & Watson, I. M. Particles in the great Pinatubo volcanic cloud of June 1991: The role of ice. Geochemistry, Geophysics, Geosystems 5, n/a-n/a, doi:10.1029/2003GC000655 (2004).

4 Kristiansen, N. I., Prata, A. J., Stohl, A. & Carn, S. A. Stratospheric volcanic ash emissions from the 13 February 2014 Kelut eruption. Geophysical Research Letters 42, 588-596, doi:10.1002/2014GL062307 (2015).

5 Nakada, S. et al. in American Geophysical Union, Fall Meeting 2014 (San Francisco, 2014).

6 Carey, S. & Sigurdsson, H. The 1982 eruptions of El Chichon volcano, Mexico (2): Observations and numerical modelling of tephra-fall distribution. Bulletin of Volcanology 48, 127-141 (1986).

7 Gutierrez-Coutino, R., Moreno-Corzo, M. & Cruz-Borraz, C. in E1 Volcan Chichonal: Proceedings of symposium during the convention of Geological Society of Mexico (Insituto de Geologia, Universidad Nacional Autonoma, 1983).

8 Schneider, D. et al. Early evolution of a stratospheric volcanic eruption cloud as observed with TOMS and AVHRR. Journal of Geophysical Research 104, 4037-4050 (1999).

9 Naranjo, J.-A., Moreno, H. & Banks, N. G. La erupcion del Volcan Hudson en 1991 (46°C), Region XI, Aisen, Chile. Bol. Serv. Nac. Geol. Min. Chile 44, 1-50 (1993).

10 Scasso, R. A., Corbella, H. & Tiberi, P. Sedimentological analysis of the tephra from the 12–15 August 1991 eruption of Hudson volcano. Bulletin of Volcanology 56, 121-132, doi:10.1007/BF00304107 (1994).

11 Constantine, E. K., Bluth, G. J. S. & Rose, W. I. in Remote Sensing of Active Volcanism 45-64 (American Geophysical Union, 2000).

12 Rybin, A. et al. Satellite and ground observations of the June 2009 eruption of Sarychev Peak volcano, Matua Island, Central Kuriles. Bulletin of Volcanology 73, 1377-1392, doi:10.1007/s00445-011-0481-0 (2011).

13 Bonadonna, C. et al. Dynamics of wind-affected volcanic plumes: The example of the 2011 Cordón Caulle eruption, Chile. Journal of Geophysical Research: Solid Earth 120, 2242-2261, doi:10.1002/2014JB011478 (2015).

14 Bignami, C. et al. Multisensor satellite monitoring of the 2011 Puyehue-Cordon Caulle eruption. IEEE journal of selected topics in applied earth observations and remote sensing 7, 2786-2796 (2014).

15 Gudmundsson, M. T. et al. in EGU General Assembly Vol. 14 (Vienna, 2012).

16 Gudnason, J. et al. in EGU General Assembly Vol. 16 (Vienna, 2014).

17 Moxnes, E. D. et al. Separation of ash and sulfur dioxide during the 2011 Grímsvötn eruption. Journal of Geophysical Research: Atmospheres 119, 7477-7501, doi:10.1002/2013JD021129 (2014).

18 Tesche, M. et al. Volcanic ash over Scandinavia originating from the Grímsvötn eruptions in May 2011. Journal of Geophysical Research: Atmospheres 117, n/a-n/a, doi:10.1029/2011JD017090 (2012).

19 Rose, W. et al. Observations of Volcanic Clouds in Their First Few Days of Atmospheric Residence: The 1992 Eruptions of Crater Peak, Mount Spurr Volcano, Alaska. The Journal of Geology 109, 677-694, doi:10.1086/323189 (2001).

20 McGimsey, R. G., Neal, C. A. & Riley, C. M. Areal distribution, thickness, mass, volume and grainsize of tephra-fall deposits from the 1992 eruptions of Creater Peak vent, Mt. Spurr Volcano, Alaska. U.S. Geological Survey Open-File Report 01-370 (2001).

21 Rose, W. I., Bluth, G. J. S. & Ernst, G. Integrating retrievals of volcanic cloud characteristics from satellite remote sensors: A summary. Phil. Trans. R. Soc. Lond. A 358, 1585-1606 (2000).

22 Wallace, K. L., Schaefer, J. R. & Coombs, M. L. Character, mass, distribution, and origin of tephra-fall deposits from the 2009 eruption of Redoubt Volcano, Alaska—Highlighting the significance of particle aggregation. Journal of Volcanology and Geothermal Research 259, 145-169, doi:http://dx.doi.org/10.1016/j.jvolgeores.2012.09.015 (2013).

23 Schmehl, K. J., Truesdell, P. A. & Haupt, S. E. in 14th Conference on Aviation, Range, and Aerospace Meteorology Vol. J11.4 (Atlanta, 2010).

24 Program, G. V. Report on Lascar (Chile). (Smithsonian Institution, 1993).

25 Viramonté, J. G., Becchio, R., Bolli, M. I., Petrinovic, I. & Tejada, R. S. A. Actividad eruptiva del volcan Lascar; erupcion 18/24 Abril 1993. (Instituto Geonorte, Nacional de Salta, Argentina, 1995).

26 Wright, R., Carn, S. A. & Flynn, L. P. A satellite chronology of the May–June 2003 eruption of Anatahan volcano. Journal of Volcanology and Geothermal Research 146, 102-116, doi:http://dx.doi.org/10.1016/j.jvolgeores.2004.10.021 (2005).

27 Trusdell, F. A. et al. The 2003 eruption of Anatahan volcano, Commonwealth of the Northern Mariana Islands: Chronology, volcanology, and deformation. Journal of Volcanology and Geothermal Research 146, 184-207, doi:http://dx.doi.org/10.1016/j.jvolgeores.2004.12.010 (2005).

28 Watt, S. F. L., Pyle, D. M., Mather, T. A., Martin, R. S. & Matthews, N. E. Fallout and distribution of volcanic ash over Argentina following the May 2008 explosive eruption of Chaitén, Chile. J. Geophys. Res. 114, B04207 (2009).

29 Rose, W. I. et al. in Volcanism and the Earth's Atmosphere 107-132 (American Geophysical Union, 2003).

30 Haraldsson, K. The Hekla 2000 eruption-distribution of ash from the first days of the eruption BSc thesis, University of Iceland, (2001).

31 Observatory, M. V. Daily Report: Report for the period 4 pm 25 September to 4 pm 26 September 1997. (1997).

32 Bonadonna, C. et al. Tephra fallout in the eruption of Soufriere Hills Volcano, Montserrat. Geological Society, London, Memoirs 21, 483-516, doi:10.1144/gsl.mem.2002.021.01.22 (2002).

33 Rose, W. I. & Mayberry, G. C. Use of GOES thermal infrared imagery for eruption scale measurements, Soufrière Hills, Montserrat. Geophysical Research Letters 27, 3097-3100, doi:10.1029/1999GL008459 (2000).

34 Turner, R. & Hurst, T. Factors Influencing Volcanic Ash Dispersal from the 1995 and 1996 Eruptions of Mount Ruapehu, New Zealand. Journal of Applied Meteorology 40, 56-69, doi:10.1175/1520-0450(2001)040<0056:fivadf>2.0.co;2 (2001).

35 Bonadonna, C. & Houghton, B. F. Total grain-size distribution and volume of tephra-fall deposits. Bulletin of Volcanology 67, 441-456, doi:10.1007/s00445-004-0386-2 (2005).

36 Prata, A. J. & Grant, I. F. Retrieval of microphysical and morphological properties of volcanic ash plumes from satellite data: Application to Mt Ruapehu, New Zealand. Quarterly Journal of the Royal Meteorological Society 127, 2153-2179, doi:10.1002/qj.49712757615 (2001).

37 Labazuy, P. et al. Near real-time monitoring of the April–May 2010 Eyjafjallajökull ash cloud: an example of a web-based, satellite data-driven, reporting system. International Journal of Environment and Pollution 48, 262-272, doi:10.1504/IJEP.2012.049673 (2012).

38 Gudmundsson, M. T. et al. Ash generation and distribution from the April-May 2010 eruption of Eyjafjallajökull, Iceland. 2, 572, doi:10.1038/srep00572 (2012).

39 Stohl, A. et al. Determination of time- and height-resolved volcanic ash emissions and their use for quantitative ash dispersion modeling: the 2010 Eyjafjallajökull eruption. Atmos. Chem. Phys. 11, 4333-4351 (2011).

40 Andronico, D., Scollo, S., Caruso, S. & Cristaldi, A. The 2002–03 Etna explosive activity: Tephra dispersal and features of the deposits. Journal of Geophysical Research: Solid Earth 113, n/a-n/a, doi:10.1029/2007JB005126 (2008).

41 Spinetti, C. et al. in 2nd MERIS/(A) ATSR User Workshop (ESA proc., 2008).
42 Carn, S. A., Strow, L. L., de Souza-Machado, S., Edmonds, Y. & Hannon, S. Quantifying tropospheric volcanic emissions with AIRS: The 2002 eruption of Mt. Etna (Italy). Geophysical Research Letters 32, n/a-n/a, doi:10.1029/2004GL021034 (2005).
43 Rose, W. I. & Schneider, D. J. Satellite images offer aircraft protection from volcanic ash clouds. Eos, Transactions American Geophysical Union 77, 529-532, doi:10.1029/96EO00345 (1996).
44 Martin-Del Pozzo, A. L., González-Morán, T., Espinasa-Pereña, R., Butron, M. A. & Reyes, M. Characterization of the recent ash emissions at Popocatepetl Volcano, Mexico. Journal of Volcanology and Geothermal Research 170, 61-75, doi:http://dx.doi.org/10.1016/j.jvolgeores.2007.09.004 (2008).
45 Corradini, S., Merucci, L. & Prata, A. J. Retrieval of SO₂ from thermal infrared satellite measurements: correction procedures for the effects of volcanic ash. Atmos. Meas. Tech. 2, 177-191, doi:10.5194/amt-2-177-2009 (2009).
46 Andronico, D., Scollo, S., Cristaldi, A. & Ferrari, F. Monitoring ash emission episodes at Mt. Etna: The 16 November 2006 case study. Journal of Volcanology and Geothermal Research 180, 123-134, doi:10.1016/j.jvolgeores.2008.10.019 (2009).
47 Corradini, S. et al. Mt. Etna tropospheric ash retrieval and sensitivity analysis using moderate resolution imaging spectroradiometer measurements. APPRES 2, 023550-023550-023520, doi:10.1117/1.3046674 (2008).
48 Maryon, R. H., Ryall, D. B. & Malcolm, A. L. The NAME 4 Dispersion Model: Science Documentation. (UK Meteorological Office, 1999).
49 Gerlach, T. M., Westrich, H. R. & Symonds, R. B. in Fire and mud: eruptions and lahars of Mount Pinatubo, Philippines (eds C. G. Newhall & R Punongbayan) 33 (Universityof Washington Press, 1996).
50 Wallace, P. J. & Gerlach, T. M. Magmatic Vapor Source for Sulfur Dioxide Released During Volcanic Eruptions: Evidence from Mount Pinatubo. Science 265, 497-499, doi:10.1126/science.265.5171.497 (1994).
51 Newhall, C. G. et al. in Fire and Mud: eruptions and lahars of Mount Pinatubo, Philippines (eds C. G. Newhall & R Punongbayan) (USGS, 1996).
52 Polacci, M., Papale, P. & Rosi, M. Textural heterogeneities in pumices from the climactic eruption of Mount Pinatubo, 15 June 1991, and implications for magma ascent dynamics. Bulletin of Volcanology 63, 83-97, doi:10.1007/s004450000123 (2001).
53 Dartevelle, S., Ernst, G. G. J., Stix, J. & Bernard, A. Origin of the Mount Pinatubo climactic eruption cloud: Implications for volcanic hazards and atmospheric impacts. Geology 30, 663-666, doi:10.1130/0091-7613(2002)030<0663:ootmpc>2.0.co;2 (2002).
54 Humaida, H., Brotopuspito, K. S., Pranowo, H. D. & narsito, n. Modelling of Magma Density and Viscocity Changes and Their Influences towards the Characteristic of Kelud Volcano Eruption. Vol. 6 (2011).
55 Caudron, C., Taisne, B., Garcés, M., Alexis, L. P. & Mialle, P. On the use of remote infrasound and seismic stations to constrain the eruptive sequence and intensity for the 2014 Kelud eruption. Geophysical Research Letters 42, 6614-6621, doi:10.1002/2015GL064885 (2015).
56 Program, G. V. Report on Kelut (Indonesia). (Smithsonian Institution, 2014).
57 Tepley, I. F. J., Davidson, J. P., Tilling, R. I. & Arth, J. G. Magma Mixing, Recharge and Eruption Histories Recorded in Plagioclase Phenocrysts from El Chichón Volcano, Mexico. Journal of Petrology 41, 1397-1411, doi:10.1093/petrology/41.9.1397 (2000).
58 Luhr, J. F., Carmichael, I. S. E. & Varekamp, J. C. The 1982 eruptions of El Chichón Volcano, Chiapas, Mexico: Mineralogy and petrology of the anhydritebearing pumices. Journal of Volcanology and Geothermal Research 23, 69-108, doi:http://dx.doi.org/10.1016/0377-0273(84)90057-X (1984).
59 Sigurdsson, H., Carey, S. N. & Espindola, J. M. The 1982 eruptions of El Chichón Volcano, Mexico: Stratigraphy of pyroclastic deposits. Journal of Volcanology and Geothermal Research 23, 11-37, doi:http://dx.doi.org/10.1016/0377-0273(84)90055-6 (1984).

60 Kratzmann, D. J., Carey, S., Scasso, R. & Naranjo, J.-A. Compositional variations and magma mixing in the 1991 eruptions of Hudson volcano, Chile. Bulletin of Volcanology 71, 419, doi:10.1007/s00445-008-0234-x (2009).
61 Scasso, R. A. & Carey, S. Morphology and formation of glassy volcanic ash from the August 12-15, 1991 eruption of Hudson Volcano, Chile. Latin American journal of sedimentology and basin analysis 12, 3-21 (2005).
62 Parejas, S., Lara, L. E., Bertin, D., Amigo, A. & Orozco, G. in EGU General Assembly Vol. 14 (Vienna, 2012).
63 Castro, J. M. et al. Storage and eruption of near-liquidus rhyolite magma at Cordón Caulle, Chile. Bulletin of Volcanology 75, 702, doi:10.1007/s00445-013-0702-9 (2013).
64 Pistolesi, M. et al. Complex dynamics of small-moderate volcanic events: the example of the 2011 rhyolitic Cordón Caulle eruption, Chile. Bulletin of Volcanology 77, 3, doi:10.1007/s00445-014-0898-3 (2015).
65 Óladóttir, B. A., Larsen, G. & Sigmarsson, O. Holocene volcanic activity at Grímsvötn, Bárdarbunga and Kverkfjöll subglacial centres beneath Vatnajökull, Iceland. Bulletin of Volcanology 73, 1187-1208, doi:10.1007/s00445-011-0461-4 (2011).
66 Sigmarsson, O. et al. The sulfur budget of the 2011 Grímsvötn eruption, Iceland. Geophysical Research Letters 40, 6095-6100, doi:10.1002/2013GL057760 (2013).
67 Liu, E. J., Cashman, K. V., Rust, A. C. & Gislason, S. R. The role of bubbles in generating fine ash during hydromagmatic eruptions. Geology 43, 239-242, doi:10.1130/g36336.1 (2015).
68 Nye, C. J., Harbin, M. L., Miller, T. P., Swanson, S. E. & Neal, C. in The 1992 eruptions of Crater Peak vent, Mount Spurr volcano, Alaska Vol. 2139 (ed Terry E.C. Keith) 119-128 (USGS, 1995).
69 Eichelberger, J., C., Keith, T. E. C., Miller, T. P. & Nye, C. J. in The 1992 eruptions of Crater Peak vent, Mount Spurr volcano, Alaska Vol. 2139 (ed Terry E.C. Keith) (USGS, 1995).
70 Gardner, C. A., Cashman, K. V. & Neal, C. A. Tephra-fall deposits from the 1992 eruption of Crater Peak, Alaska: implications of clast textures for eruptive processes. Bulletin of Volcanology 59, 537-555, doi:10.1007/s004450050208 (1998).
71 Miller, T. P., Neal, C. A. & Waitt, R. B. in The 1992 eruptions of Crater Peak vent, Mount Spurr volcano, Alaska. USGS Bulletin 2139 (ed Terry E.C. Keith) 81-88 (USGS, 1995).
72 Coombs, M. L. et al. Andesites of the 2009 eruption of Redoubt Volcano, Alaska. Journal of Volcanology and Geothermal Research 259, 349-372, doi:http://dx.doi.org/10.1016/j.jvolgeores.2012.01.002 (2013).
73 Werner, C. et al. Degassing of CO2, SO2, and H2S associated with the 2009 eruption of Redoubt Volcano, Alaska. Journal of Volcanology and Geothermal Research 259, 270-284, doi:http://dx.doi.org/10.1016/j.jvolgeores.2012.04.012 (2013).
74 Matthews, S. J., Gardeweg, M. C. & Sparks, R. S. J. The 1984 to 1996 cyclic activity of Lascar Volcano, northern Chile: cycles of dome growth, dome subsidence, degassing and explosive eruptions. Bulletin of Volcanology 59, 72-82, doi:10.1007/s004450050176 (1997).
75 Matthews, S. J., Jones, A. P. & Gardeweg, M. C. Lascar Volcano, Northern Chile; Evidence for Steady-State Disequilibrium. Journal of Petrology 35, 401-432, doi:10.1093/petrology/35.2.401 (1994).
76 Calder, E. S., Sparks, R. S. J. & Gardeweg, M. C. Erosion, transport and segregation of pumice and lithic clasts in pyroclastic flows inferred from ignimbrite at Lascar Volcano, Chile. Journal of Volcanology and Geothermal Research 104, 201-235, doi:http://dx.doi.org/10.1016/S0377-0273(00)00207-9 (2000).
77 Wade, J. A. et al. The May 2003 eruption of Anatahan volcano, Mariana Islands: Geochemical evolution of a silicic island-arc volcano. Journal of Volcanology and Geothermal Research 146, 139-170, doi:https://doi.org/10.1016/j.jvolgeores.2004.11.035 (2005).
78 Pallister, J. S. et al. The 2003 phreatomagmatic eruptions of Anatahan volcano—textural and petrologic features of deposits at an emergent island volcano. Journal of Volcanology and Geothermal Research 146, 208-225, doi:https://doi.org/10.1016/j.jvolgeores.2004.11.036 (2005).

79 Nakada, S. et al. Geological aspects of the 2003–2004 eruption of Anatahan Volcano, Northern Mariana Islands. Journal of Volcanology and Geothermal Research 146, 226-240, doi:https://doi.org/10.1016/j.jvolgeores.2004.10.023 (2005).

80 Castro, J. M. & Dingwell, D. B. Rapid ascent of rhyolitic magma at Chaiten volcano, Chile. Nature 461, 780-783, doi:http://www.nature.com/nature/journal/v461/n7265/suppinfo/nature08458_S1.html (2009).

81 Höskuldsson, Á. et al. The millennium eruption of Hekla in February 2000. Bulletin of Volcanology 70, 169-182, doi:10.1007/s00445-007-0128-3 (2007).
Iceland. Earth and Planetary Science Letters 255, 373-389, doi:http://dx.doi.org/10.1016/j.epsl.2006.12.024 (2007).

83 Rutherford, M. J. & Devine, J. D. Magmatic Conditions and Magma Ascent as Indicated by Hornblende Phase Equilibria and Reactions in the 1995–2002 Soufrière Hills Magma. Journal of Petrology 44, 1433-1453, doi:10.1093/petrology/44.8.1433 (2003).

84 Edmonds, M., Pyle, D. & Oppenheimer, C. A model for degassing at the Soufrière Hills Volcano, Montserrat, West Indies, based on geochemical data. Earth and Planetary Science Letters 186, 159-173, doi:https://doi.org/10.1016/S0012-821X(01)00242-4 (2001).

85 Druitt, T. H. et al. in The Eruption of SoufriOre Hills Volcano, Montserrat, from 1995 to 1999. Vol. 21 (eds T. H. Druitt & Peter Kokelaar) 281-306 (Geological Society, 2002).

86 Kilgour, G., Blundy, J., Cashman, K. & Mader, H. M. Small volume andesite magmas and melt–mush interactions at Ruapehu, New Zealand: evidence from melt inclusions. Contrib Mineral Petrol 166, 371-392, doi:10.1007/s00410-013-0880-7 (2013).

87 Nakagawa, M., Wada, K., Thordarson, T., Wood, C. P. & Gamble, J. A. Petrologic investigations of the 1995 and 1996 eruptions of Ruapehu volcano, New Zealand: formation of discrete and small magma pockets and their intermittent discharge. Bulletin of Volcanology 61, 15-31, doi:10.1007/s004450050259 (1999).

88 Sigmarsson, O. et al. Remobilization of silicic intrusion by mafic magmas during the 2010 Eyjafjallajökull eruption. Solid Earth 2, 271-281, doi:10.5194/se-2-271-2011 (2011).

89 Moune, S., Sigmarsson, O., Schiano, P., Thordarson, T. & Keiding, J. K. Melt inclusion constraints on the magma source of Eyjafjallajökull 2010 flank eruption. Journal of Geophysical Research: Solid Earth 117, n/a-n/a, doi:10.1029/2011JB008718 (2012).

90 Sigmundsson, F. et al. Intrusion triggering of the 2010 Eyjafjallajokull explosive eruption. Nature 468, 426-430, doi:http://www.nature.com/nature/journal/v468/n7322/abs/nature09558.html#supplementary-information (2010).

91 Dellino, P. et al. Ash from the Eyjafjallajökull eruption (Iceland): Fragmentation processes and aerodynamic behavior. Journal of Geophysical Research: Solid Earth 117, n/a-n/a, doi:10.1029/2011JB008726 (2012).

92 Cioni, R. et al. Insights into the dynamics and evolution of the 2010 Eyjafjallajökull summit eruption (Iceland) provided by volcanic ash textures. Earth and Planetary Science Letters 394, 111-123, doi:10.1016/j.epsl.2014.02.051 (2014).

93 Spilliaert, N., Métrich, N. & Allard, P. S–Cl–F degassing pattern of water-rich alkali basalt: Modelling and relationship with eruption styles on Mount Etna volcano. Earth and Planetary Science Letters 248, 772-786, doi:http://dx.doi.org/10.1016/j.epsl.2006.06.031 (2006).

94 Andronico, D., Cristaldi, A., Del Carlo, P. & Taddeucci, J. Shifting styles of basaltic explosive activity during the 2002–03 eruption of Mt. Etna, Italy. Journal of Volcanology and Geothermal Research 180, 110-122, doi:10.1016/j.jvolgeores.2008.07.026 (2009).

95 Straub, S. M. & Martin-Del Pozzo, A. L. The significance of phenocryst diversity in tephra from recent eruptions at Popocatepetl volcano (central Mexico). Contrib Mineral Petrol 140, 487-510, doi:10.1007/pl00007675 (2001).

96 Atlas, Z. D., Dixon, J. E., Sen, G., Finny, M. & Martin-Del Pozzo, A. L. Melt inclusions from Volcán Popocatépetl and Volcán de Colima, Mexico: Melt evolution due to vapor-saturated crystallization during ascent. Journal of Volcanology and Geothermal Research 153, 221-240, doi:http://dx.doi.org/10.1016/j.jvolgeores.2005.06.010 (2006).

97 Costa, A., Pioli, L., & Bonadonna, C. (2016). Assessing tephra total grain-size distribution: Insights from field data analysis. *Earth and Planetary Science Letters*, *443*, 90-107.
